# Absence of Testes at Puberty Impacts Functional Development of Nigrostriatal But Not Mesoaccumbal Dopamine Terminals in a Wild-Derived Mouse

**DOI:** 10.1523/ENEURO.0212-25.2025

**Published:** 2026-01-07

**Authors:** Samantha Jackson, Jaewan Mun, George Prounis, Chayarndorn Phumsatitpong, Niloofar Motahari, Lance Kriegsfeld, Markita P. Landry, Linda Wilbrecht

**Affiliations:** ^1^Department of Neuroscience, University of California, Berkeley, Berkeley, California 94720; ^2^California Institute for Quantitative Biosciences, University of California, Berkeley, Berkeley, California 94720; ^3^Departments of Psychology, University of California, Berkeley, Berkeley, California 94720; ^4^Molecular and Cell Biology, University of California, Berkeley, Berkeley, California 94720; ^5^Chemical & Biomolecular Engineering, University of California, Berkeley, Berkeley, California 94720

**Keywords:** adolescence, dopamine system, gonadectomy, *Mus spicilegus*, nIRCats, striatum

## Abstract

The nigrostriatal and mesoaccumbal dopamine systems are thought to contribute to changes in behavior and learning during adolescence, yet it is unclear how the rise in gonadal hormones at puberty impacts the function of these systems. We studied the impact of prepubertal gonadectomy (GDX) on later evoked dopamine release in male *Mus spicilegus*, a mouse whose adolescent life history has been carefully characterized in the wild and laboratory. To examine how puberty impacts dopamine neuron function in *M. spicilegus* males, we removed the gonads prepubertally at postnatal day (P)25 and then examined evoked dopamine release in the dorsomedial, dorsolateral (DLS), and nucleus accumbens core regions of striatal slices at P60–70 (late adolescence/early adulthood). To measure dopamine release, we used near-infrared catecholamine nanosensors which enable study of spatial distribution of dopamine release. We found that prepubertal GDX led to a significantly reduced density of dopamine release sites and reduced dopamine release at each site in the DLS nigrostriatal system compared with sham controls. In contrast, mesoaccumbal dopamine release was comparable between sham and gonadectomized groups. Our data suggest that during adolescence, the development of the nigrostriatal dopamine system is significantly affected by the rise in gonadal hormones in males, while the mesoaccumbal system shows no detectable sensitivity at this time point. These data are consistent with molecular studies in rodents that suggest nigrostriatal neurons are sensitive to androgens at puberty and extend our understanding of how gonadal hormones could impact the spatial distribution and release potential of dopamine terminals in the striatum.

## Significance Statement

Here we use a wild-derived species, *Mus spicilegus*, to study adolescent development. This species has value over standard lab mice because it is more likely to exhibit evolved developmental programs relevant to dispersal and other natural behaviors. By using this wild-derived species and metrics of evoked dopamine release with spatial resolution, we can test if the rise in gonadal hormones at puberty plays a role in maturation of dopamine terminal function in the striatum. These findings may help us better understand developmental programs in humans that orchestrate changes in behavior at adolescent milestones in contexts of both health and disease.

## Introduction

Across many species, adolescence is a time of increased exploration, sensation-seeking, and adaptive and sometimes maladaptive risk-taking (Dahl et al., 2018; Worthman and Trang, 2018; Natterson-Horowitz and Bowers, 2020; Cryns et al., 2022; Lin and Wilbrecht, 2022). These behavioral changes are accompanied by significant structural and functional remodeling of the brain, including changes in dopamine neurons and their terminals in the striatum (Larsen et al., 2020), as well as the neocortex (Hoops and Flores, 2017; Delevich et al., 2021). Changes in mammalian dopamine systems during adolescence are of particular interest because they may be implicated in changes in learning and decision-making as well as the development of mental health issues that emerge in adolescence (Ge et al., 2001; Graber et al., 2004; Deardorff et al., 2007; Mendle et al., 2018; Copeland et al., 2019; Simpson et al., 2022).

Here we focus on male *Mus spicilegus* mice and the development of dopamine in the nigrostriatal and mesoaccumbal pathways of the dopamine system. Several studies suggest nigrostriatal and/or mesoaccumbal dopamine neurons show dynamic changes across adolescent development in rodent brains. Multiple studies in rats show evoked dopamine release increases with development in males (Gazzara et al., 1986; Stamford, 1989; Pitts et al., 2020), but some show a decrease (Kuhn et al., 2010). Sex and regional differences can also differ, making consensus difficult.

Once rodents reach adulthood, tyrosine hydroxylase (TH) levels in nigrostriatal and mesoaccumbal neurons and the striatal tissue are sensitive to activational effects of gonadal hormones, which can be isolated by examining the effects of gonadectomy (GDX; i.e., castration) followed by hormone replacement (Abreu et al., 1988). The regulation of terminal release of dopamine in the striatum has also been found to be modulated by testosterone after GDX (Shemisa et al., 2006). However, not all results again agree or show a consistent direction of effect (Xiao and Becker, 1994). The effects of gonadal hormones on cells at puberty can have opposing effects in adulthood. For example, male rats that undergo GDX in adulthood exhibit a decrease in cocaine-stimulated dopamine “overflow” in the dorsal striatum, but rats that undergo GDX prior to puberty can exhibit an increase in dopamine “overflow” (Kuhn et al., 2010). In summary, there is a strong body of evidence suggesting gonadal hormones in males play a role in midbrain dopamine neuron development, but there are still open questions about the role of gonadal hormones at the time of puberty and their impact on terminal growth and function.

To clarify the role of gonadal hormones in dopamine neuron development, we studied the effect of prepubertal GDX (P25–28) on evoked dopamine release in the striatal dorsomedial (DMS), dorsolateral (DLS), and nucleus accumbens (NAc) regions in male *Mus spicilegus* during late adolescence/early adulthood (P60–70) using near-infrared catecholamine sensors (nIRCats). nIRCats are synthetic optical reporters built by functionalizing single-walled carbon nanotubes (SWCNTs) using single-stranded (GT)_6_ oligonucleotides to enable the SWCNTs to fluoresce in the presence of catecholamines (Beyene et al., 2019). Because the striatum is densely innervated by nigrostriatal and mesoaccumbal dopaminergic neurons and lacks innervation from neurons that release norepinephrine, it enables study of evoked dopamine release in this region (Beyene et al., 2019). Here, we chose to use nIRCats because they allow for optical imaging of evoked dopamine release sites at two-dimensional “hotspots” in a brain slice, giving us more granular information than previously available about terminal function and density (Beyene et al., 2019). We opted to study a wild-derived species, *M. spicilegus*, rather than standard laboratory mice, to identify ethologically relevant developmental programs that may link maturation of dopamine neurons to pubertal changes. *M. spicilegus* is a particularly valuable species to study because their adolescent behavior has been documented in the wild. For example, when born during the spring/summer in the wild, these mice are known to disperse from their natal sites within their first 3 months of life. However, when born during the fall/winter, this dispersal behavior is delayed until the following spring (Garza et al., 1997; Gouat et al., 2003; Poteaux et al., 2008; Simeonovska-Nikolova and Mehmed, 2009). *M. spicilegus* also show developmental changes in increased risk-taking and exploratory behavior in the lab during this adolescent to adult transition period (Groó et al., 2013; Lafaille and Féron, 2014; Cryns et al., 2022; Bárdos et al., 2024). We therefore expect if there are programs that couple dopamine system maturation to pubertal processes that we will have (1) a high likelihood of detecting them in this wild-derived species, and (2) the significance of these behavioral changes may be interpretable in an ethological context.

## Materials and Methods

### Animals

Male *M. spicilegus* were bred in-house. Mice were weaned on Postnatal Day (P)21 and group-housed on a 12:12 h light/dark cycle. Animals were given access to food and water *ad libitum* and housed with nesting material. Mice underwent GDX or sham surgery between P25 and 28. Between P60 and 70, mice were perfused, and brains were collected. All procedures were approved by the Animal Care and Use Committee of the University of California, Berkeley and conformed to principles outlined by the NIH Guide for the Care and Use of Laboratory Animals.

### Synthesis of nIRCats

High-pressure carbon monoxide-synthesized SWCNTs and (GT)_6_ oligonucleotides (Beyene et al., 2019) were purchased from NanoIntegris and Integrated DNA Technologies, respectively. A 0.1 mM of (GT)_6_ and 1 mg of SWCNT were mixed in 1 ml of 100 mM NaCl solution. The mixture was first bath-sonicated (Branson Ultrasonic 1800) and then probe-tip sonicated (Cole-Parmer ultrasonic processor, 3 mm tip in diameter, 5 W power) in an ice water bath for 10 min each. The resulting suspension was centrifuged at 16,800 × *g* for 90 min to remove unsuspended SWCNT aggregates. The supernatant was collected for further purification. To remove excessive free (GT)_6_ in the recovered suspension, Amicon spin filter tubes (MW 100k) were used. A 500 μl of the suspension was spin-filtered at 8,000 × *g* for 5 min, and the filtrate was discarded. A 500 μl of molecular-grade water was added to the spin filter tube and was centrifuged at 8,000 × *g* for 5 min. This wash step with water was repeated three times. To recover concentrated (GT)_6_–SWCNT suspension, the filter tube was reversed into a new bottom container and spun at 1,000 × *g* for 5 min. The final sample was collected and used for characterization.

### Characterization of nIRCats

The concentration of the collected (GT)_6_–SWCNT was characterized by its absorbance at 632nm, which was measured by Nanodrop (Thermo Fisher Scientific). An extinction coefficient of 0.036 L mg^−1^ cm^−1^ at 632 nm was used. Each batch of nIRCats was then diluted to 5 mg L^−1^ in 0.1 M NaCl for in vitro fluorescence measurements. A99 μl (GT)_6_–SWCNT was placed in a well of a 384-well plate. Fluorescence spectra were collected with an inverted Zeiss microscope (20× objective lens, Axio Observer D1) coupled to a spectrometer (SCT 320, Princeton Instruments) and a liquid nitrogen-cooled InGaAs detector (PyLoN-IR, Princeton Instruments). A 721 nm laser (Opto Engine LLC) was used as an excitation light source. To observe the response of nIRCats to dopamine, we measured the fluorescence before and after 1 μl of 100× diluted dopamine hydrochloride (Sigma-Aldrich) was added to the nanosensor. nIRCats increase in fluorescence by up to 3,500% in the presence of catecholamines.

### GDX

To examine adolescent development in the absence of steroid sex hormones, GDX surgeries were performed before puberty onset between P25 and 28 as described previously (Delevich et al., 2020). Before surgery, mice were injected with analgesics 0.05 mg/kg buprenorphine and 10 mg/kg meloxicam and anesthetized with 1–2% isoflurane during surgery. The incision area was shaved and scrubbed with ethanol and betadine. Ophthalmic ointment was applied over the eyes to prevent drying. A 1 cm incision was made with a scalpel in the lower abdomen across the midline to access the abdominal cavity. The testes were clamped off from the spermatic cord with locking forceps and ligated with sterile sutures. After ligation, the gonads were excised with a scalpel. The muscle and skin layers were sutured, and wound clips were placed over the incision for 7–10 d to allow the incision to heal. An additional injection of buprenorphine was given 12 h later and meloxicam 24 and 48 h later. Sham control surgeries were identical to GDX except that the gonads were simply visualized and were not clamped, ligated, or excised. Mice were allowed to recover on a heating pad until ambulatory and were monitored daily to check for normal weight gain and to monitor for signs of discomfort/distress. Mice were cohoused with 1–2 siblings who received the same surgical treatment. Subjects in this study included six intact sham controls and six castrated (GDX) males.

### Acute brain slice preparation and nanosensor incubation

Acute brain slices were prepared from 12:12 h reared mice aged between P60 and 70, which we interpret to be a late adolescent/cusp of adulthood transition period in this species when raised on this photoperiod (Cryns et al., 2022). Briefly, mice were deeply anesthetized via isoflurane exposure, followed by an overdose of ketamine/xylazine solution. Transcardial perfusion was performed by injecting ice-cold cutting buffer (in mM: 119 NaCl, 26.2 NaHCO_3_, 2.5 KCl, 1 NaH_2_PO_4_, 3.5 MgCl_2_, 10 glucose, and 0 CaCl_2_). The 300-μm-thick coronal slices were cut in ice-cold cutting solution before being incubated in ACSF buffer (in mM: 119 NaCl, 26.2 NaHCO_3_, 2.5 KCl, 1 NaH_2_PO_4_, 1.3 MgCl_2_, 10 glucose, and 2 CaCl_2_). Slices were bubbled with 95% O_2_/5% CO_2_ at 37°C for 30 min and then incubated at room temperature. Slices were incubated in a small incubation chamber (Scientific Systems Design) with 2 mg L^−1^ nIRCats in 5 ml ACSF buffer bubbling with 95% O_2_/5% CO_2_ for 15 min. The slices were washed with ACSF buffer to remove unlabeled nIRCats and kept in ACSF buffer for another 15 min before imaging.

### nIRCat imaging in ex vivo slices using nIR microscopy

Dopamine imaging was performed ex vivo with a modified upright epifluorescent microscope. Details of the microscope are described in a previous study (Beyene et al., 2019). Excitation of nIRCats was done via a 785 nm laser, and fluorescence was measured with an InGaAs detector (Ninox 640). A slice labeled with nIRCats was transferred onto the sample stage of the microscope. The surface of the slice was made in contact with a bipolar stimulating electrode using 4× and 60× objective lenses. Specifically, the stimulator was positioned next to the imaging field of view in DMS, DLS, or NAc. All stimulation experiments were recorded with a frame rate of 9 frames per second and single-pulse electrical stimulations were applied after 200 frames of the baseline were acquired. Each video acquisition lasted 600 frames. Within a field of view, stimulation amplitudes were repeated three times, with 5 min in between for terminal recovery.

### Data analysis of nIRCat fluorescence response

A python-based image analysis tool was used to process a raw 600-frame image stack (Ouassil, 2022). First, a field of view was divided into multiple regions of interest (ROIs) 7 × 7 μm in size, and “active release sites” were defined as ROIs that showed a significant change in fluorescence over the background after electrical stimulation. Specifically, if the fluorescence change in a given region exceeded three times the standard deviation of baseline fluctuations, it was considered active, and based on the sensor and location, we infer it was dopamine-induced (Beyene et al., 2019). Release site density was calculated by dividing the number of active ROIs by the total number of ROIs in the field of view.

In each ROI, Δ*F*/*F*_0_ was achieved by dividing the change of fluorescence by the baseline. Next, time constants of signal onset (τ_on_) and decay (*τ*_off_) were extracted by approximating the Δ*F*/*F*_0_ versus time curve as a product of exponential rise/decay functions as follows:

$$\frac{dF}{F}=A\;\times \;\left(1-\;\exp\;\left(-\frac{t}{\tau_{\mathrm{on}}}\right)\right)\times \;\exp\;\left(-\frac{t}{\tau_{\mathrm{off}}}\right)+\;B.$$

Here, *A* and *B* are constants. Active release site density, peak Δ*F*/*F*_0_, and *τ*_off_ were compared between GDX and sham. Each parameter was calculated for a single field of view averaging three replicates (or two replicates if stimulation electrode lost contact with slice on one of the three replicates). For *τ*_off_, values were excluded if the fitted *τ*_off_ value exceeded 10 s, as this provided *τ*_off_ values that closely followed the decay curve of the overall field of view fluorescence.

As a control for our 7 × 7 μm grid ROI analysis methods, we additionally investigated the impact of changing the ROI grid size on our three major metrics: active release site density, Δ*F*/*F*_0_ per active release site, and *τ*_off_ using ROI sizes of 4 × 4 μm and 10 × 10 μm (to compare to our 7 × 7 μm method used in the main manuscript). We found that reducing the ROI size to 4 × 4 μm did not significantly impact data or comparison of GDX to sham groups (Extended Data Figs. 1-1, 1-2*a*–*c*). However, the signal-to-noise ratio was reduced when the ROI size was increased to 10 × 10 μm, resulting in fewer active ROIs in some regions and issues with fitting the fluorescence response and extracting *τ*_off_ from some slices. These changes reduce overall average metrics by introducing zero values for some slices (Extended Data Fig. 1-2*c*). These data inform the utility of the smaller 7 × 7 μm ROI choice which was selected to be large enough to contain 95% of the evoked “hotspot” sizes observed in previous work (Beyene et al., 2019).

### Fecal testosterone collection and analysis

Fecal matters (0.4 g) were collected from home cages of group-housed animals reared on a 12:12 h light/dark cycle every 10 d from P20 to 60. All mice were placed in new cages the day before collection to minimize steroid decay due to old fecal samples. Fecal samples were stored at −20°C until processed. Prior to testosterone extraction, fecal samples were dried and ground into a powder. Drying was performed by placing the samples in an oven at 65°C for a maximum of 90 min. The dried samples were then ground into a fine powder using a coffee grinder and transferred to 1.5 ml Eppendorf tubes using a funnel. The tubes were subsequently weighed to adjust the sample weight to 0.2 g, with precise weights recorded for all samples. Steroids were extracted following the Steroid Solid Extraction protocol from Arbor Assays. Briefly, 0.2 g of dried fecal material was mixed with 1.8 ml of ethanol and shaken vigorously for 30 min. The samples were then concentrated by air-dry in the oven at 65°C and resuspended in 100 μl of ethanol. The concentrated extract was then diluted further with Assay Buffer. Fecal testosterone was determined in duplicate aliquots using the DetectX testosterone enzyme immunoassay kit (Arbor Assays). The intra-assay coefficient of variation for this assay was 8.48%. Assay sensitivity was 9.92 pg/ml.

### Statistics

We compared metrics of dopamine release between sham and GDX by fitting a linear mixed-effect model with restricted maximum likelihood to the data. For Figures 2–4, surgical treatment was held as the fixed effect, and the animal sampled was held as the random effect. We report *t* statistic, *p* value, and effect size using Cohen's *f*^2^. For Figure 5, the striatal region was held as the fixed effect and the animal sampled as the random effect. We performed a post hoc analysis with Tukey's test on the pairwise comparisons between the striatal regions, reporting a *z*-score for each comparison. We used *η*^2^_*p*_ as a measure of effect size of the model, and Hedges’ *g* as a measure of the effect size for each pairwise comparison. For Extended Data Figure 1-2, the ROI size was held as the fixed effect and the animal sampled as the random effect. We also performed similar analyses as Figure 5, reporting the *z*-score from Tukey's post hoc test, *η*^2^_*p*_ as the model's effect size, and Hedges’ *g* as the effect size for the pairwise comparisons between the ROI sizes. For Extended Data Figure 1-1, we performed similar analyses as Figures 2–4 but with ROI sizes 4 × 4 μm and 10 × 10 μm. The “nlme” package was used for linear mixed-effect modeling and “multcomp” for Tukey's post hoc testing (RStudio). We compared metrics of dopamine release across the DMS, DLS, and NAc in each treatment group with a repeated-measure correlation in Extended Data Figure 5-1 using the “rmcorr” package, reporting the *r_m_* correlation coefficient as a measure of the effect size (RStudio). We used code from Möller GitHub repository (Möller, 2025) to calculate Cohen's *f*^2^ in RStudio. The package “effectsize” was used to calculate *η*^2^_*p*_ and “esvis” to calculate Hedges’ *g* (RStudio). Mean ± standard error of the mean (SEM) was calculated for each group, unless otherwise noted. Statistical significance is indicated as * for *p* < 0.05, ** for *p* < 0.01, and *** for *p* < 0.001. See Extended Data Statistical Table 1-1 for full statistical reporting.

## Results

**Figure 1.**
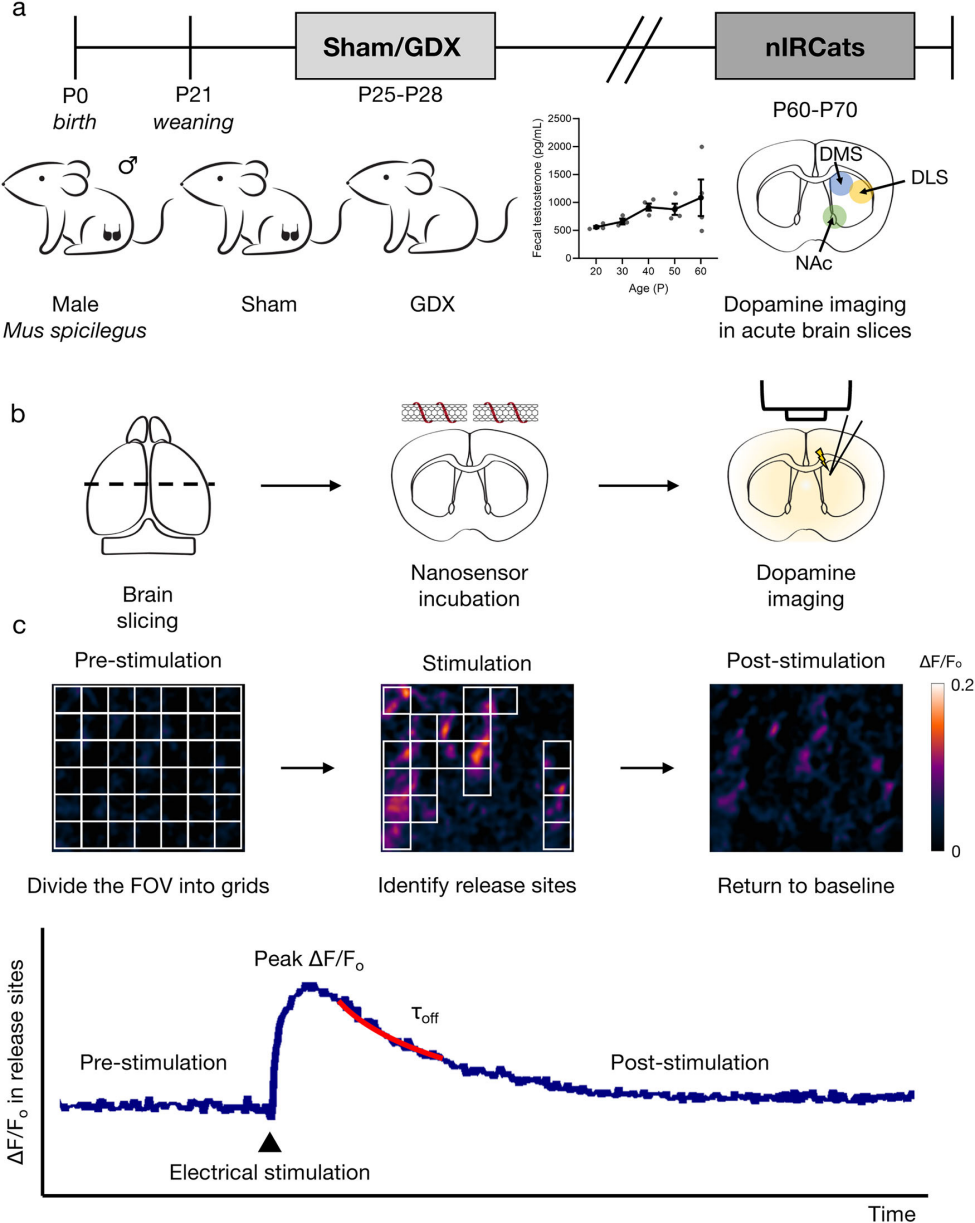
Schematic of the experimental timeline using nIRCat nanosensor based technology. ***a***, Male *M. spicilegus* were gonadectomized (GDX) after weaning at P25–28 to remove gonads before onset of puberty. Data from a separate cohort of male *M. spicilegus* show an increase in average fecal testosterone from P20 to 60 with high variability at P60 [*n* = 4 cages, each cage (with multiple individuals) is shown as an individual gray dot]. Error bars represent the SEM. Dopamine imaging in acute brain slices from sham/GDX mice was performed during late adolescence or cusp of early adulthood (P60–P70) in this species reared on this 12:12 h light cycle. ***b***, Schematic of imaging experiments in brain slices. Acute brain slices were prepared and labeled with dopamine nanosensors. Electrically evoked dopamine release was monitored in nIR with a custom-built microscope. ***c***, Schematic of data analysis for nanosensor fluorescence response in brain slices. Three metrics of dopamine release (density of active ROIs as fraction of total ROIs, peak Δ*F*/*F* within active ROIs, and *τ*_off_) were extracted from the nIRCat fluorescence response and used for comparison between groups. Extended Data Figures 1-1 and 1-2 show that using smaller and larger ROI grid sizes produced comparable results.

After weaning at P21, male *M. spicilegus* were gonadectomized at P25–28 or underwent sham surgery as a control (Fig. 1*a*). Age at GDX was chosen based on data from *Mus musculus* (Piekarski et al., 2017) and a preliminary study of fecal testosterone (T) of *M. spicilegus* in which we found a ∼50% rise in T between P30 and 90 with high variability between cages (Fig. 1*a*). Reasoning from these hormonal data, a behavioral study from our lab (Cryns et al., 2022), and previous literature suggesting *M. spicilegus* males reach sexual maturity between P60 and 80 (Gouat et al., 2003; Lafaille et al., 2015), we estimate P60–70 is a late adolescent or cusp of early adulthood period in this species when reared on 12:12 h photoperiod in the lab environment.

10.1523/ENEURO.0212-25.2025.f1-1 Figure 1-1 **Group differences between treatment groups were replicated using a smaller 4** **×** **4 μm ROI grid size than 7** **×** **7 μm**, **while some group differences were no longer significant using a larger 10** **×** **10 μm ROI grid size.** When the ROI grid size was set at 4 × 4 μm resolution comparisons of evoked dopamine release, release site density, and τ_off_ replicated the results obtained applying a 7 × 7 μm resolution grid (see results in the main manuscript). However, when ROI sizes were set at 10 × 10 μm resolution some group differences were no longer significant (DMS peak ΔF/F_0_ and DLS τ_off_). Changes with larger grid size may be due to loss of detection of some signals when averaged with non-responsive pixels within the ROI. Linear mixed effects model and effect size calculated using Cohen’s *f*^2^. *p < 0.05. Download Figure 1-1, DOCX file.

**Figure 2.**
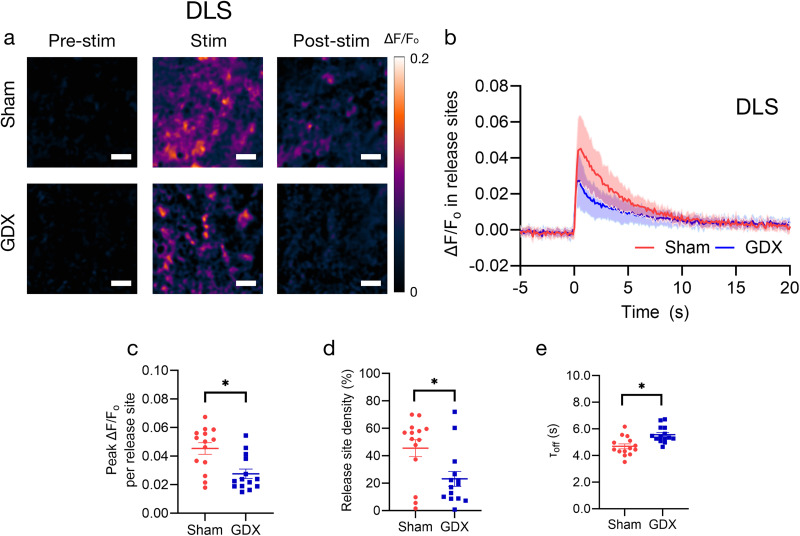
The density of dopamine release sites and release at each site was lower in the DLS of adult mice that underwent prepubertal GDX compared with sham controls. (***a***) Repeat images of the same field of view in the DLS and Δ*F*/*F*_0_ of nIRCat signal in sham (top) and GDX (bottom) mice. “Pre-stim” is before electrical stimulation is applied, “Stim” is the peak Δ*F*/*F*_0_ following stimulation, and “Post-stim” is when fluorescence is returning to the baseline. ***b***, Average change in Δ*F*/*F*_0_ in the DLS in response to 0.3 mA electrical stimulation applied at 0 s in sham (*n* = 14 brain slices/6 mice) and GDX (*n* = 14 brain slices/6 mice) mice. Change in fluorescence reflects electrically evoked dopamine release at time 0. The shaded area represents standard deviation. Metrics of dopamine release based on nIRCat signal per slice are summarized in ***c–e***. ***c***, Average peak Δ*F*/*F*_0_ at active release sites (defined as 7 µm squares ROIs where release was at least three times the standard deviation of baseline fluctuation after electrical stimulation). ***d***, Active release site density (the percentage of total sites in FOV that were defined as active) and (***e***) *τ*_off_ calculated using Δ*F*/*F*_0_ from only active release sites (active 7 × 7 µm ROIs). Scale bars, 10 µm. *p < 0.05. Error bars indicate the SEM.

10.1523/ENEURO.0212-25.2025.f1-2 Figure 1-2 **Evoked dopamine release, release site density, and reuptake related metric were comparable across regions and treatment groups when ROI sizes were set at smaller resolution.** Comparison of (a) peak ΔF/F_0_ per release site, (b) release site density, and (c) τ_off_ between ROI sizes of 7 × 7 μm, 4 × 4 μm, and 10 × 10 μm for each region. Plots of data from all mice (a-c) showed few significant changes in mean data values when analyzed using three different grid sizes (4, 7, 10 μm). Linear mixed effects model followed by post-hoc analysis with Tukey’s test. **p < 0.01. Error bars represent the SEM. Download Figure 1-2, TIF file.

10.1523/ENEURO.0212-25.2025.t1-2 Table 1-2 Statistical Table. Download Figure 1-2, DOCX file.

To examine the effect of prepubertal GDX on the function of the nigrostriatal and mesoaccumbal dopamine system at this P60–70 stage, acute brain slices were prepared following previously reported protocols (Beyene et al., 2019; Yang et al., 2021; Fig. 1*b*). Next, we imaged electrically evoked nIRCat responses in three striatal regions: the DLS, the DMS, and the NAc using a custom-built microscope with a near-infrared (nIR) camera. Dopamine release was evoked using a 1 ms single electrical pulse (0.3 mA) from a bipolar stimulating electrode, and Δ*F*/*F*_0_ was calculated for all pixels with nIRCat labeling. We divided each field of view (178 × 142 μm) into 546 ROIs (7 × 7 μm in size) and measured the Δ*F*/*F*_0_ for each ROI (Fig. 1*c*). In our previous study, we observed that electrically evoked dopamine diffuses into the surrounding extracellular space, with over 95% of release sites exhibiting diffusion restricted within 7 μm (Beyene et al., 2019). A grid size of 7 μm was therefore chosen to capture the relevant spatial extent of dopamine diffusion. Additional control experiments (Extended Data Fig. 1-1) showed smaller and larger grid sizes produced comparable results.

We identified ROIs with significant fluorescence modulation following stimulation and designated them as dopamine release sites (active ROIs). Specifically, if the fluorescence change in a given region after stimulation exceeded three times the standard deviation of baseline fluctuations, it was considered evoked dopamine-induced. Since nIRCats fluorescence modulation is dopamine-dependent, the fraction of active ROIs serves as a proxy for dopamine release site density (assuming there is no evoked norepinephrine release in the striatum; Beyene et al., 2019). Next, we analyzed the change of Δ*F*/*F*_0_ at active release sites over time to characterize dopamine release decay dynamics after stimulation (Fig. 1*c*).

### Prepubertal GDX males showed fewer and weaker active dopamine release sites in the dorsal striatum than sham controls

We found that a single pulse of electrical stimulation induced clear fluorescence modulation of nIRCats in the DLS (Fig. 2*a*). The fluorescence of nIRCats returned to the baseline following an exponential decay poststimulation, enabling the use of the same field of view for subsequent stimulations (Fig. 2*a*,*b*). Overall, GDX mice exhibited significantly smaller evoked dopamine release upon electrical stimulation in the DLS than the sham cohort. When comparing GDX and sham groups in metrics of density of active release sites (defined here as active 7 × 7 μm ROIs) and average peak Δ*F*/*F*_0_ in each release site, we found that GDX mice showed a significantly lower density of active release sites (expressed as a percentage of total sites; Fig. 2*d*; sham 45.543 ± 6.186% vs GDX 23.171 ± 5.467%; *t*_(10)_ = 2.315; *p*^b^ = 0.043*; *f*^2^ = 0.206) and significantly smaller average peak Δ*F*/*F*_0_ in each of these release sites (active ROIs only; Fig. 2*c*; sham 0.045 ± 0.004 vs GDX 0.028 ± 0.003; *t*_(10)_ = 2.427; *p*^a^ = 0.036*; *f*^2^ = 0.226). Of course, dopamine released from terminals upon electrical stimulation is a function of both the density of terminals and release per terminal. Our results suggest that prepubertal GDX affected both the density of terminals, as measured by percentage density of active sites, and approximate release per terminal, as measured by peak Δ*F*/*F*_0_ per active site in the DLS.

Next, we compared the decay of the evoked signal which may reflect dopamine reuptake dynamics. We quantified this reuptake feature using an exponential decay function (Figs. 1*c*, 2*b*). The *τ*_off_ was extracted from the curve to measure the decay time from peak fluorescence back to the baseline. We found that GDX mice exhibited larger *τ*_off_ [sham 4.694 ± 0.188 s(s) vs GDX 5.570 ± 0.162 s; *t*_(10)_ = −2.821; *p*^c^ = 0.018*; *f*^2^ = 0.306; Fig. 2*e*], suggesting that a lack of gonadal hormones at puberty may also block an increase in dopamine reuptake in the DLS seen in late adolescence/early adulthood.

**Figure 3.**
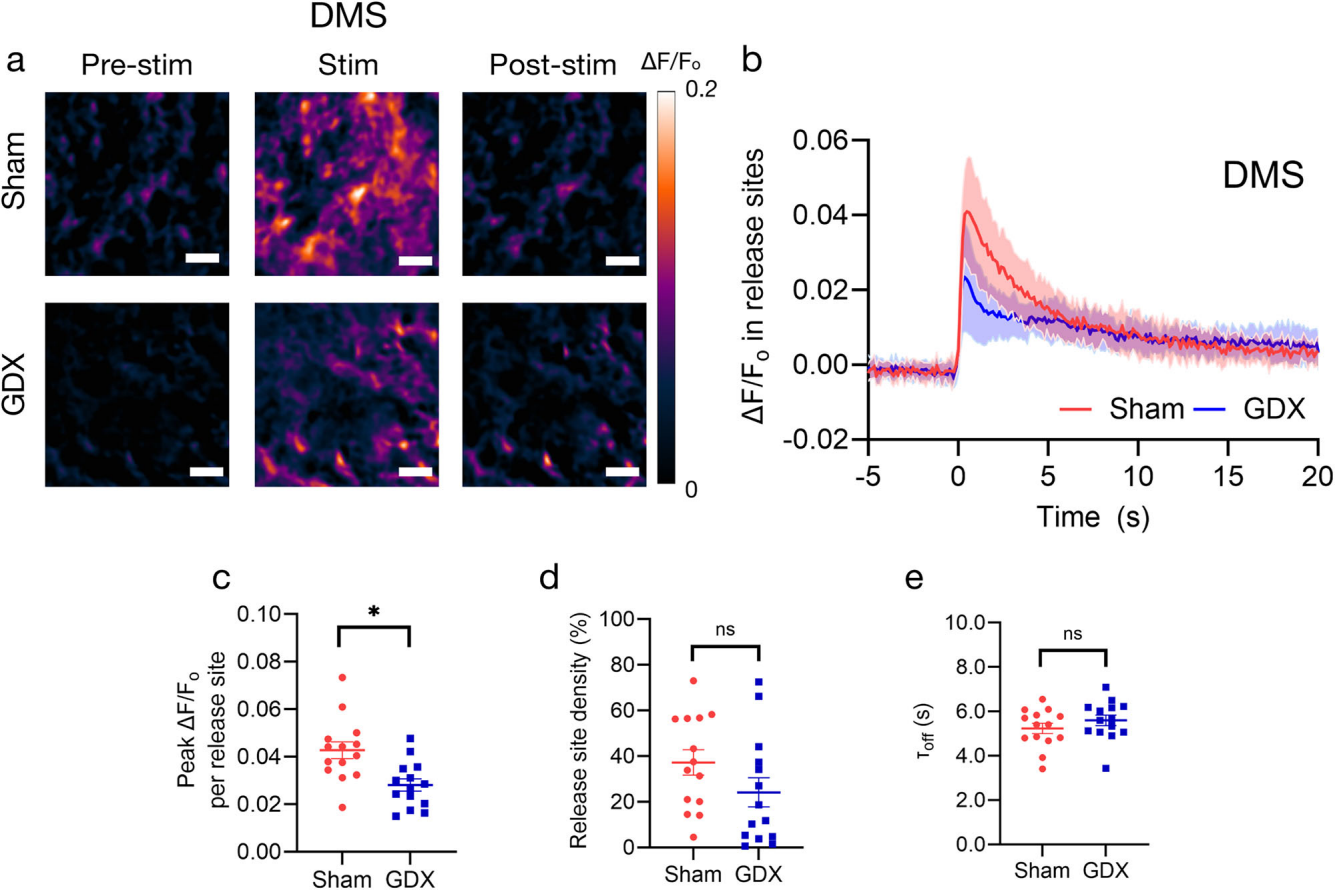
Dopamine release at active sites was lower in the DMS of adult mice that underwent prepubertal GDX compared with sham controls. (***a***) Repeat images of the same field of view in the DMS and Δ*F*/*F*_0_ of nIRCat signal in sham (top) and GDX (bottom) mice. “Pre-stim” is before electrical stimulation is applied, “Stim” is the peak Δ*F*/*F*_0_ following stimulation, and “Post-stim” is when fluorescence is returning to the baseline. ***b***, Δ*F*/*F*_0_ change in DMS in response to 0.3 mA electrical stimulation applied at 0 s in sham (*n* = 14 brain slices/6 mice) and GDX mice (*n* = 14 brain slices/6 mice). Change in fluorescence reflects electrically evoked dopamine release at time 0. The shaded area represents standard deviation. Metrics of dopamine release based on nIRCat signal per slice are summarized in ***c–e***. ***c***, Average peak Δ*F*/*F*_0_ at active release sites (defined as 7 × 7 µm square ROIs where release was at least three times the standard deviation of baseline fluctuation after electrical stimulation). ***d***, Active release site density (the percentage of total FOV defined as active) and (***e***) *τ*_off_ calculated using Δ*F*/*F*_0_ from only active release sites (active 7 µm ROIs). Scale bars, 10 µm. *p < 0.05. Error bars indicate the SEM.

We next investigated the DMS, as it is well known that the DLS and DMS, while both targets of nigrostriatal dopamine, play different roles in behavior (Yin and Knowlton, 2006). In the DMS, as in the DLS, the GDX group showed significantly reduced peak Δ*F*/*F*_0_ per active release site compared with the sham group (sham 0.043 ± 0.004 vs GDX 0.028 ± 0.003; *t*_(10)_ = 2.309; *p*^d^ = 0.044*; *f*^2^ = 0.205; Fig. 3*a*–*c*). However, in the DMS, no significant difference was observed in the density of active release sites (sham 37.229 ± 5.549% vs GDX 24.164 ± 6.364%; *t*_(10)_ = 1.356; *p*^e^ = 0.205; *f*^2^ = 0.071; Fig. 3*d*). This result suggests that in the DMS, the pubertal rise in gonadal hormones in males may not significantly affect the number of dopamine release sites but does influence the amount of dopamine released per release site. In the DMS, we found no significant difference in *τ*_off_ (sham 5.240 ± 0.234 s vs GDX 5.601 ± 0.238 s; *t*_(10)_ = −0.807; *p*^f^ = 0.439; *f*^2^ = 0.025), suggesting dopamine reuptake in DMS was not sensitive to gonadal hormones at puberty (Fig. 3*e*).

### Prepubertal GDX had little impact on evoked dopamine release in the ventral striatum

**Figure 4.**
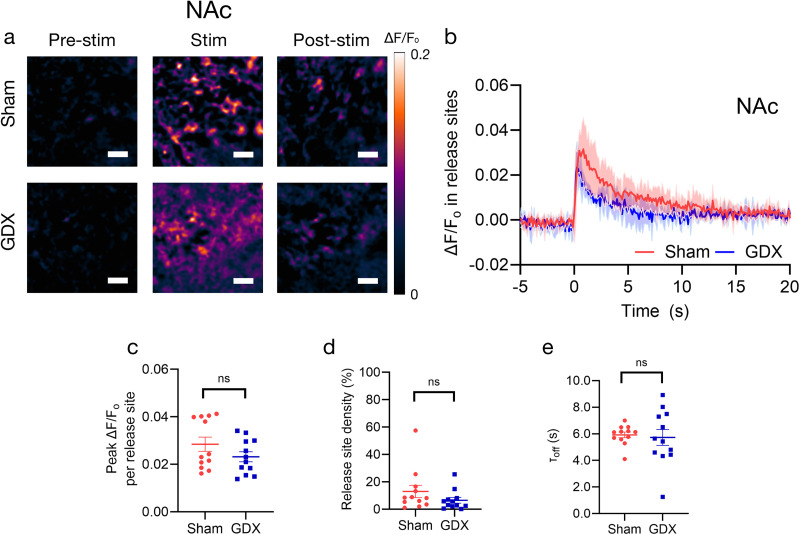
Evoked dopamine in the NAc at P60 was not significantly affected by prepubertal GDX in *M. spicilegus.* ***a***, Repeat images of a field of view in NAc and Δ*F*/*F*_0_ of nIRCat signal before, following, and after electrical stimulation in sham (top) and GDX (bottom) mice. “Pre-stim” is before electrical stimulation is applied, “Stim” is the peak Δ*F*/*F*_0_ following stimulation, and “Post-stim” is when fluorescence is returning to the baseline. ***b***, Δ*F*/*F*_0_ change in NAc in response to 0.3 mA electrical stimulation applied at 0 s in sham (*n* = 12 brain slices/5 mice) and GDX mice (*n* = 12 brain slices/6 mice). Change in fluorescence reflects electrically evoked dopamine release at time 0. The shaded area represents standard deviation. Metrics of dopamine release based on nIRCat signal in each slice are summarized in ***c–e***. ***c***, Average peak Δ*F*/*F*_0_ at active release sites (defined as 7 µm squares ROIs where release was at least three times the standard deviation of baseline fluctuation after electrical stimulation). ***d***, Active release site density (the percentage of total FOV defined as active) and (***e***) *τ*_off_ calculated using Δ*F*/*F*_0_ from only active release sites (active 7 µm ROIs). Scale bars, 10 µm. *p < 0.05. Error bars indicate the SEM.

**Figure 5.**
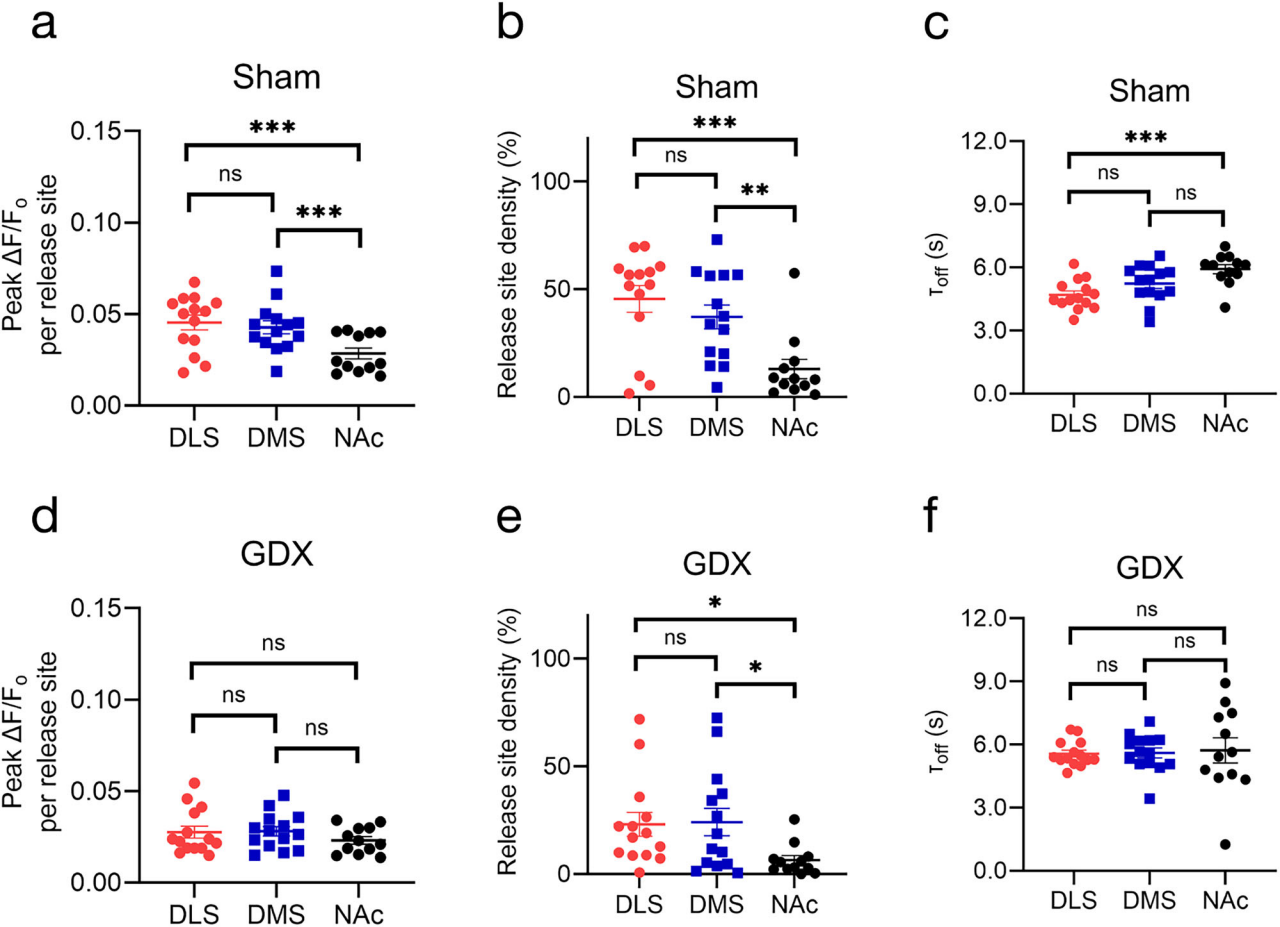
Prepubertal GDX eliminates regional differences in dorsal versus ventral evoked dopamine release, release site density, and reuptake. Comparison of metrics from sham (***a–c***) and GDX (***d–f***) mice. ***a***, ***d***, Comparison of peak Δ*F*/*F*_0_ per release site between DMS, DLS, and NAc of (***a***) sham and (***d***) GDX mice shows dorsal versus ventral differences in sham that are not apparent in GDX. ***b***, ***e***, Comparison of release site density between the DMS, DLS, and NAc reveals dorsal versus ventral differences in sham (***b***) that are only partially observed in GDX mice (***e***). ***c***, ***f***, Comparison of *τ*_off_ between the DMS, DLS, and NAc reveals DLS versus NAc difference in sham (***c***) that is not observed in GDX mice (***f***). Linear mixed-effect model followed by post hoc analysis with Tukey's test. *p < 0.05; **p < 0.01; ***p < 0.001. Error bars indicate the SEM. Extended Data Figure 5-1 show correlations between release site density and Δ*F*/*F*_0_ per active release site in dorsal striatum but not NAc.

Finally, we examined dopamine release in the NAc core in the same mice used above. Dopaminergic projections to the ventral striatum (VS), including the NAc, originate from the ventral tegmental area (VTA) in the midbrain. This projection is also called the mesoaccumbal dopamine pathway. We found that GDX and sham mice showed comparable metrics of evoked dopamine release when quantified for the whole field of view (Fig. 4*b*), Δ*F*/*F*_0_ per active release site (sham 0.028 ± 0.003 vs GDX 0.023 ± 0.002; *t*_(9)_ = 1.448; *p*^g^ = 0.182; *f*^2^ = 0.095; Fig. 4*c*), and density of release sites (sham 13.058 ± 4.498% vs GDX 6.55 ± 2.076%; *t*_(9)_ = 1.314; *p*^h^ = 0.221; *f*^2^ = 0.078; Fig. 4*d*). The *τ*_off_ decay time constants were also comparable (sham 5.922 ± 0.213 s vs GDX 5.728 ± 0.600 s; *t*_(9)_ = 0.305; *p*^i^ = 0.767; *f*^2^ = 0.004; Fig. 4*e*). These data suggest that gonadal hormones at puberty in male *M. spicilegus* mice had little impact on the mesoaccumbal dopamine system terminals at P60–70. To study similarities and differences between striatal regions with sham and GDX groups, we also plotted metrics of dopamine release side-by-side in new panels. We then compared metrics of dopamine release across the DMS, DLS, and NAc within the group using a linear mixed-effect model followed by post hoc analysis with Tukey's test. We found that, in sham males, dopamine release was significantly greater in the dorsal striatum (DMS and DLS) compared with the NAc due to greater release site density [*z* = −3.131; *p*^o^ = 0.005**; *g* = −1.26 (NAc − DMS); *z* = −4.199; *p*^n^ = 0.00007***; *g* = −1.57 (NAc − DLS)] and greater evoked Δ*F*/*F*_0_ per site [*z* = −3.695; *p*^l^ = 0.0007***; *g* = −1.15 (NAc − DMS); *z* = −4.297; *p*^k^ = 0.00005***; *g* = −1.23 (NAc − DLS)]. In sham, there were no significant differences observed between medial and lateral dorsal striatum [*z* = −1.116; *p*^m^ = 0.504; *g* = −0.367 (DMS − DLS release site density); *z* = −0.635; *p*^j^ = 0.801; *g* = −0.179 (DMS − DLS Δ*F*/*F*_0_); Fig. 5*a*,*b*]. A significant difference in the reuptake time constant was observed between the DLS and NAc [*z* = 4.159; *p*^q^ = 0.0001***; *g* = 1.66 (NAc − DLS)] but not other regions [*z* = 2.329; *p*^r^ = 0.052; *g* = 0.810 (NAc − DMS); *z* = 1.912; *p*^p^ = 0.135; *g* = 0.667 (DMS − DLS); Fig. 5*c*]. Within the striatum of GDX males, release site density was higher in the dorsal striatum (DMS and DLS) than in the NAc [*z* = −2.488; *p*^w^ = 0.034*; *g* = −1.02 (NAc − DLS); *z* = −2.633; *p*^x^ = 0.023*; *g* = −0.938 (NAc − DMS); *z* = 0.152; *p*^v^ = 0.987; *g* = 0.043 (DMS − DLS); Fig. 5*e*], but Δ*F*/*F*_0_ per active release site and *τ*_off_ at active sites in DMS and DLS were comparable to NAc [*z* = −1.383; *p*^t^ = 0.350; *g* = −0.423 (NAc − DLS Δ*F*/*F*_0_); *z* = −1.518; *p*^u^ = 0.282; *g* = −0.552 (NAc − DMS Δ*F*/*F*_0_); *z* = 0.141; *p*^s^ = 0.989; *g* = 0.040 (DMS − DLS Δ*F*/*F*_0_); *z* = 0.300; *p*^z^ = 0.952; *g* = 0.104 (NAc − DLS *τ*_off_); *z* = 0.238; *p*^ab^ = 0.969; *g* = 0.079 (NAc − DMS *τ*_off_); *z* = 0.064; *p*^y^ = 0.998; *g* = 0.040 (DMS − DLS *τ*_off_); Fig. 5*d*,*f*].

10.1523/ENEURO.0212-25.2025.f5-1 Figure 5-1 **Evoked dopamine release and release site density were positively correlated in the dorsal striatum, but not NAc.** (a) Comparison between peak ΔF/F_0_ per release site and release site density, (b) peak ΔF/F_0_ per release site and τ_off_, and (c) τ_off_ and release site density for each region and treatment group. Repeated measures correlation. *p < 0.05. These analyses were exploratory and *p*-values were not corrected for multiple comparisons. Download Figure 5-1, TIF file.

In a final exploration of our data, we tested for correlations between release site density, Δ*F*/*F*_0_ per active release site, and *τ*_off_ obtained from each slice in each region and treatment group. We found positive correlations between release site density and Δ*F*/*F*_0_ per active release site in dorsal striatum (DMS and DLS) for both sham and GDX groups [*r_m_* = 0.786; *p*^ac^ = 0.012* (DLS sham); *r_m_* = 0.698; *p*^ad^ = 0.037* (DMS sham); *r_m_* = 0.761; *p*^af^ = 0.017* (DLS GDX); *r_m_* = 0.769; *p*^ag^ = 0.016* (DMS GDX); reporting uncorrected *p* values; Extended Data Fig. 5-1*a*]. No significant correlation between these metrics was observed in the NAc in either sham or GDX group [*r_m_* = 0.223; *p*^ae^ = 0.596 (sham); *r_m_* = 0.235; *p*^ah^ = 0.613 (GDX); Extended Data Fig. 5-1*a*]. Measures of Δ*F*/*F*_0_ per active release site and *τ*_off_ were significantly negatively correlated in DMS in the sham group (*r_m_* = −0.689; *p*^aj^ = 0.040*), but no other significant correlations were observed between *τ*_off_ and other metrics in sham or GDX group [Δ*F*/*F*_0_ per active release site vs *τ*_off_, *r_m_* = 0.382; *p*^ai^ = 0.310 (DLS sham); *r_m_* = −0.185; *p*^ak^ = 0.661 (NAc sham); *r_m_* = −0.588; *p*^al^ = 0.096 (DLS GDX); *r_m_* = −0.112; *p*^am^ = 0.774 (DMS GDX); *r_m_* = −0.540; *p*^an^ = 0.211 (NAc GDX); *τ*_off_ vs release site density, *r_m_* = 0.259; *p*^ao^ = 0.501 (DLS sham); *r_m_* = −0.430; *p*^ap^ = 0.248 (DMS sham); *r_m_* = 0.051; *p*^aq^ = 0.905 (NAc sham); *r_m_* = −0.454; *p*^ar^ = 0.220 (DLS GDX); *r_m_* = −0.043; *p*^as^ = 0.913 (DMS GDX); *r_m_* = −0.305; *p*^at^ = 0.506 (NAc GDX); Extended Data Fig. 5-1*b*,*c*].

## Discussion

We prepared acute brain slices from male *M. spicilegus* mice raised on long-day photoperiod to assess the influence of gonadal hormones at puberty on evoked dopamine release in the DMS, DLS, and NAc. We were interested in characterizing this species because its adolescent behaviors have been documented in the wild and its natural developmental programs for dispersal and other behaviors are less likely to be disrupted by long-term laboratory breeding. We found that the absence of testes at puberty impacted evoked dopamine release in the nigrostriatal dopamine system, but not the mesoaccumbal system.

Using nIRCats enabled us to evaluate the impact of GDX on evoked dopamine release at terminals with new spatial resolution with 7 × 7 μm ROIs. This spatial analysis revealed that in the DLS, the fraction of total ROIs that were active after stimulation was significantly smaller in GDX mice compared with sham controls and *τ*_off_ was longer. Also, within these active ROIs in both the DLS and DMS, the peak of evoked nIRCat signals was significantly lower in GDX mice when compared with sham controls. In the NAc, nIRCat signals in active ROIs were comparable between GDX and controls. These data support a working model in which a rise in gonadal hormones at puberty, likely testosterone or one of its metabolites, drives an expansion of nigrostriatal terminal density as well as a gain of function in release from individual terminals, without impacting mesoaccumbal terminal function (at least at this stage). The data also suggest that pubertal hormones enhance dopamine reuptake in the DLS, but not in other regions.

Our experiments have a number of limitations. We did not replace hormones in adulthood so we cannot distinguish between organizational or activational effects of gonadal steroids. We also did not study female mice so we cannot say if puberty plays a similar or different role in female development. Given the limitations of the single age sampled, we also cannot rule out the possibility of a situation in which complex responses to the GDX manipulation caused a diminishment in terminal release density and reuptake in the nigrostriatal system compared with sham controls. Additionally, the smaller sample size for the NAc group compared with the DLS and DMS groups could have limited the power to detect effects. However, based on other literature, we favor the hypothesis that gonadal steroids in males at puberty drives enhancement in function of nigrostriatal terminals.

The published literature, largely using laboratory rats for experiments, establishes that the substantia nigra pars compacta (SNpc) and VTA neurons that make dopamine are sensitive to gonadal steroids. Both estrogen and androgen receptors are expressed in nigrostriatal SNpc neurons in the midbrain (Quesada et al., 2007; Johnson et al., 2010; Kuhn et al., 2010; Purves-Tyson et al., 2012; Sárvári et al., 2014). The VTA mesoaccumbal system expresses estrogen receptors as well but may have fewer androgen receptors and/or they may not be expressed in dopamine neurons (Kritzer, 1997). Furthermore, in male rats, studies of adolescent GDX and comparison of GDX rats treated with testosterone, dihydrotestosterone, or estradiol suggest SNpc neurons can upregulate their TH and dopamine transporter (DAT) mRNA expression in response to androgens but not estrogens during adolescence (Purves-Tyson et al., 2014). Interestingly, hormone-driven changes in TH and DAT were significant in soma samples of SNpc neurons but not significant in the striatal tissue when sampling from their terminals (Purves-Tyson et al., 2014). This is not consistent with our data, but it is possible nIRCat imaging is simply a more sensitive metric to detect a change at terminals. Based on Purves-Tyson et al. (2014), we favor a model in which activation of androgen receptors in the SNpc is the mechanism by which puberty supports trophic changes in nigrostriatal dopamine release. This model can be falsified or supported in future experiments.

As we establish a mechanistic understanding of the development of the dopamine system by linking it to hormone receptor types and exploring changes at the level of terminal density, release, and reuptake, we can better inform our understanding of developmental changes in exploratory behavior, motivation, salience, and learning during adolescence. This knowledge may provide mechanistic insights into understanding human adolescent vulnerability to anxiety and depression. Data from C57 Bl/6 mice show prepubertal GDX in male mice, but not female mice, reduces exploratory behavior in the elevated plus maze and enhances learned helplessness in the forced swim task (Boivin et al., 2017; Delevich et al., 2020). These are rodent tasks considered relevant to human anxiety and depression and together with our current data suggest gonadal hormones at puberty play a prominent role in male mice in the development of bold and persistent behaviors, possibly through nigrostriatal dopamine.

Viewing these physiological and behavioral studies of prepubertal GDX from a neuroethological perspective may help us understand how pubertal processes could drive changes in behavior like dispersal, territory identification, and/or mate seeking (Wright et al., 2020; Lin and Wilbrecht, 2022). Our data are taken from *M. spicilegus*, a wild-derived mouse known to disperse from their natal sites if they are born in the spring and delay their dispersal until the following spring if they are born in the winter (Garza et al., 1997; Gouat et al., 2003; Poteaux et al., 2008; Simeonovska-Nikolova and Mehmed, 2009; Cryns et al., 2022). Our mice were reared on 12:12 h photoperiod in the lab which may mimic spring/summer light and to drive earlier maturation more in line with laboratory rodent life history. These data suggest a potential underlying mechanism for driving adolescent dispersal behavior, namely, a rise in testosterone driving a gain in function in dopamine release from nigrostriatal terminals. As their adolescent life history is seasonal, we are interested in testing this idea in future studies by investigating the development of the dopamine system in age-matched cohorts of *M. spicilegus* reared on short-day photoperiods.

Our data are taken from ex vivo slice and so of course cannot fully capture functional changes that may be occurring across puberty in vivo. Neurons in the VTA and striatum have been shown to change their firing properties across adolescence. In anesthetized rats in vivo, VTA neurons were found to fire ∼40% faster in adolescents than adults and have higher burst duration, greater number of spikes per burst, and shorter postburst recovery period (McCutcheon et al., 2012). In awake-behaving rats, dorsal striatal neurons were, as a population, more active in adolescent rats than adults during a behavioral task; however, no difference in activity between age groups was observed in the VS (Sturman and Moghaddam, 2012). Further recording work in vivo will be needed to examine how dopamine release changes with age and puberty in a task context and how this affects activity of specific downstream neurons. Developmental changes in dopamine in the NAc shell and posterior tail of the striatum may also be worthy of investigation because these areas are also implicated in exploratory behavior (Nicola, 2010; Morrison et al., 2017; Menegas et al., 2018; Akiti et al., 2022; Tsutsui-Kimura et al., 2025).

In conclusion, our data show that dopamine terminal function, particularly in the DLS striatum, is altered by gonadal hormones at puberty in the male mouse brain. These data are only a piece of a larger puzzle, but they contribute to a growing body of evidence that the striatum does continue to develop during puberty (Larsen et al., 2020). This protracted maturation may have implications for striatal contribution to natural behaviors, which can be fruitfully explored in *M. spicilegus*. These results may also help us to identify new mechanisms that play a potential role in the etiology of mental health issues in human adolescence.

## Synthesis

Reviewing Editor: Nathalie Ginovart, University of Geneva Decisions are customarily a result of the Reviewing Editor and the peer reviewers coming together and discussing their recommendations until a consensus is reached. When revisions are invited, a fact-based synthesis statement explaining their decision and outlining what is needed to prepare a revision will be listed below. The following reviewer(s) agreed to reveal their identity: Brian Trainor, Kuan Wang. Note: If this manuscript was transferred from JNeurosci and a decision was made to accept the manuscript without peer review, a synthesis may not be available.

This manuscript was reviewed by two experts in the field. Overall, it was felt that this is a promising and well-conceived study that offers novel insights into how pubertal hormones shape dopamine system development in a non-traditional model species. With revisions to better frame the rationale, explain the techniques, and address key statistical and interpretational issues, the manuscript will make a valuable contribution to the field.

Editor:

Per e-Neuro policy, a statistical table is required that contains full statistical reporting for all analyses throughout the manuscript.

The detailed reviewer comments are as follows:

Reviewer 1:

In the introduction the authors should provide some more detail what was known about the timing of puberty in this species (similar to Mus, delayed?). If it was minimal the authors should mention they are filling that gap in the present manuscript. There is quite a lot of variability at P60 for testosterone levels. Does this track with other puberty markers?

What is the direction of developmental changes in risk-taking and exploratory behavior.

Since nIRCat is a catecholamine sensor that can't distinguish between dopamine and norepinephrine, the authors should explain how they know they are performing dopamine imaging. Is it possible that these mice could have norepinephrine release in the brain regions studied?

The discussion has a lot of rehashing what was done or found. I suggest the authors focus more on what these results mean.

Discussion of aromatization should consider the distribution of aromatase in the brain. In mammals aromatase expression is low comparted to other vertebrates and concentrated in a few (mostly hypothalamic/limbic) brain regions. The authors should check whether there are significant amounts of aromatase activity in midbrain (my guess is there is not much there).

Reviewer 2:

This manuscript investigates how prepubertal gonadectomy impacts dopamine release in adolescent male Mus spicilegus, with particular focus on the nigrostriatal and mesoaccumbal pathways. The authors employ an elegant combination of a novel model organism and cutting-edge optical nanosensors (nIRCats) to probe region-specific dopaminergic dynamics in striatal slices. The study reveals that prepubertal removal of gonads attenuates dopamine release and release site density in the dorsolateral striatum, while apparently sparing the nucleus accumbens core. These findings contribute meaningfully to our understanding of how pubertal hormones shape the development of dopaminergic circuits.

The study is methodologically innovative and tackles a timely question in developmental neurobiology. However, the manuscript would benefit from improvements in framing the rationale for species selection, elaboration on the measurement technique, clarification of statistical reporting and interpretation, and more careful contextualization of the developmental timeline in M. spicilegus. Below are specific comments and recommendations.

1. Rationale for Model Organism

The choice of Mus spicilegus should be justified more clearly in the Introduction. Currently, its unique behavioral and developmental features (e.g., seasonality, photoperiod sensitivity) are not introduced until the Discussion (Lines 344-352). Clarify early on how these traits might provide insights into puberty-dependent modulation of DA systems would be helpful.

2. Explanation of nIRCat Technology

The manuscript should more clearly describe the working principles and advantages of nIRCats in the Introduction, beyond citing prior work. What does this method measure? How does it compare to other methods? Acronyms like HiPco SWCNTs should be spelled out and explained in the Methods section for broader accessibility.

3. Validation of Dopamine Specificity

Have previous studies validated that nIRCat signal changes in the striatum are specific to dopamine? This point is critical and should be addressed either in the Introduction or Methods and revisited in the Discussion to bolster confidence in interpretation.

4. Potential DA release and Tau_off Correlations

In Figure 5, there appears to be a relationship between reduced release site density, peak dF/F, and increased tau_off. If individual-level correlations exist across slices or mice, these should be quantified and interpreted.

5. Developmental Timing Clarity

The manuscript should clearly define the timing of puberty and adolescence in M. spicilegus. While P25-28 are treated as prepubertal, and P60-70 are described as either 'late adolescence' (Line 68) or 'adult' (Line 139), these classifications should be supported with developmental data or appropriate citations.

Minor Points: Consider reorganizing the Discussion so that references to photoperiod (Line 279-280) and ecological traits (Lines 344-352) of M. spicilegus are foregrounded and more clearly linked to the experimental findings.

Statistics:

Effect Sizes and P-values: The manuscript should report effect sizes and exact p-values (not just thresholds), particularly for comparisons in Figures 3d, 4c, and 4d. Given similar trends of GDX effects across striatal regions, reliance on a p < 0.05 threshold may not robustly support the binary conclusion that mesoaccumbal circuits are unaffected. Particularly, the n for NAc is smaller than for DLS and DMS, potentially limiting power to detect effects. A discussion of this limitation should be included.

Figure 1a Variability: The SEM for P60 is particularly large. Plotting individual cage means (4 cages in total) would help readers assess biological variability. The source of this heterogeneity should be discussed.

Image Analysis Software:

Based on the method description, the choice of a 7 μm × 7 μm grid to measure release sites needs justification. Was this resolution optimized empirically or chosen based on prior studies? Could alternative scales change the interpretation of release density?

## Author Response

Response to Reviewers Our responses to specific comments are in line below in bold. Editor Comment:

Per e-Neuro policy, a statistical table is required that contains full statistical reporting for all analyses throughout the manuscript.

Extended Data Statistical Table added in submission with the manuscript.

Reviewer 1:

In the introduction the authors should provide some more detail what was known about the timing of puberty in this species (similar to Mus, delayed?). If it was minimal the authors should mention they are filling that gap in the present manuscript. There is quite a lot of variability at P60 for testosterone levels. Does this track with other puberty markers? Currently, not much is known about the timing of puberty in Mus spicilegus. We now reason more about the stage they are at using T and behavioral data (see response to next comment) and have now added more detail about the adolescent life history of this species. We write: "For example, when born during the spring/summer in the wild, these mice are known to disperse from their natal sites within their first three months of life. However, when born during the fall/winter, this dispersal behavior is delayed until the following spring (Garza et al., 1997; Gouat et al., 2003; Poteaux et al., 2008; Simeonovska-Nikolova and Mehmed, 2009). M. spicilegus also show developmental changes in increased risk-taking and exploratory behavior in the lab during this adolescent to adult transition period (Groó et al., 2013; Lafaille and Féron, 2014; Cryns et al., 2022; Bárdos et al., 2024)." (Lines 83 - 90) We acknowledge there is great variability in our preliminary study of fecal testosterone levels in these animals. Because the T was so variable and we do not have other puberty marker data, we rely on our T data plus behavioral data from our lab and others to estimate the stage of mice. We now write in the text: "Age at gonadectomy was chosen based on data from Mus musculus (Piekarski et al., 2017) and a preliminary study of fecal testosterone (T) of M. spicilegus in which we found a ~ 50% rise in T between P30-90 with high variability between cages (Figure 1a). Reasoning from these hormonal data, a behavioral study (Cryns et al., 2022) and previous literature suggesting M. spicilegus males reach sexual maturity between P60-80 (Gouat et al., 2003; Lafaille et al., 2015), we estimate P60-70 is a late adolescent or cusp of early adulthood period in this species when reared on 12h:12h photoperiod in the lab environment." (Lines 254 - 261) What is the direction of developmental changes in risk-taking and exploratory behavior.

In the Introduction, we added that changes in risk-taking and exploratory behavior increase across adolescence. "Across many species, adolescence is a time of increased exploration, sensation- seeking, and adaptive and sometimes maladaptive risk-taking (Dahl et al., 2018; Worthman and Trang, 2018; Natterson-Horowitz and Bowers, 2020; Cryns et al., 2022; Lin and Wilbrecht, 2022)." (Lines 34 - 37) Since nIRCat is a catecholamine sensor that can't distinguish between dopamine and norepinephrine, the authors should explain how they know they are performing dopamine imaging. Is it possible that these mice could have norepinephrine release in the brain regions studied? In the Introduction, we added that because the striatum lacks innervation from neurons that release norepinephrine and is densely innervated by dopaminergic terminals from the substantia nigra and ventral tegmental areas, we know that we are measuring evoked dopamine release in these regions. "Because the striatum is densely innervated by nigrostriatal and mesoaccumbal dopaminergic neurons and lacks innervation from neurons that release norepinephrine, it enables study of evoked dopamine release in this region (Beyene et al., 2019)." (Lines 73 - 76) The discussion has a lot of rehashing what was done or found. I suggest the authors focus more on what these results mean.

We elaborated more on the interpretation of our results in the second paragraph of the Discussion (Lines 387 - 398).

Discussion of aromatization should consider the distribution of aromatase in the brain. In mammals, aromatase expression is low compared to other vertebrates and concentrated in a few (mostly hypothalamic/limbic) brain regions. The authors should check whether there are significant amounts of aromatase activity in midbrain (my guess is there is not much there).

Thanks for this nudge. After reviewing the literature, we found a few papers that suggest that aromatization activity mainly occurs in the neonate mouse midbrain, but once the mouse reaches adulthood, there is a lack of aromatization in the midbrain. Stanić et al. does find aromatase in the dorsal striatum and NAc shell, but it is not showing where we imaged. Given we cannot be confident about regional expression, species similarity, or what happens to expression in peri-puberty, we opted to remove the following statement from our Discussion: "Testosterone from the testes could act on SNpc neurons by binding to androgen receptors, but it could also be converted to 17-beta estradiol through aromatization in the brain." Supporting literature we consulted for reference:

Ivanova T, Beyer C (2003) Estrogen regulates tyrosine hydroxylase expression in the neonate mouse midbrain. J Neurobiol 54(4):638-47.

MacLusky NJ, Walters MJ, Clark AS, Toran-Allerand CD (1994) Aromatase in the cerebral cortex, hippocampus, and mid-brain: ontogeny and developmental implications. Mol Cell Neurosci 5(6):691-8.

Stanić D, Dubois S, Chua HK, Tonge B, Rinehart N, Horne MK, Boon WC (2014) Characterization of aromatase expression in the adult male and female mouse brain. I.

Coexistence with oestrogen receptors α and β, and androgen receptors. PLoS One 9(3):e90451.

Thanks for Rev 1 time and energy reading our manuscript! Reviewer 2:

1. Rationale for Model Organism The choice of Mus spicilegus should be justified more clearly in the Introduction. Currently, its unique behavioral and developmental features (e.g., seasonality, photoperiod sensitivity) are not introduced until the Discussion (Lines 344-352). Clarify early on how these traits might provide insights into puberty-dependent modulation of DA systems would be helpful.

Thanks for bringing this up. Indeed, we want to celebrate these mice. We have now added more detail about Mus spicilegus in the Introduction. "We opted to study a wild-derived species, Mus spicilegus, rather than standard laboratory mice, to identify ethologically relevant developmental programs that may link maturation of dopamine neurons to pubertal changes. Mus spicilegus is a particularly valuable species to study because..." (Lines 79 - 93) 2. Explanation of nIRCat Technology The manuscript should more clearly describe the working principles and advantages of nIRCats in the Introduction, beyond citing prior work. What does this method measure? How does it compare to other methods? Acronyms like HiPco SWCNTs should be spelled out and explained in the Methods section for broader accessibility.

We apologize for this oversight. In the Introduction, we now address better what nIRCats are, what they measure, and how they compare to other methods. We also spelled out the acronyms in the Methods. "nIRCats are synthetic optical reporters built by functionalizing single-walled carbon nanotubes (SWCNTs) using single-stranded (GT)6 oligonucleotides to enable the SWCNTs to fluoresce in the presence of catecholamines (Beyene et al., 2019)." (Lines 70 - 73) "Here, we chose to use nIRCats because they allow for optical imaging of evoked dopamine release sites at 2-dimensional "hotspots" in a brain slice, giving us more granular information than previously available about terminal function and density (Beyene et al., 2019)." (Lines 76 - 79) "High-pressure carbon monoxide (HiPco)-synthesized single-walled carbon nanotubes (SWCNTs)..." (Lines 104 - 105) 3. Validation of Dopamine Specificity Have previous studies validated that nIRCat signal changes in the striatum are specific to dopamine? This point is critical and should be addressed either in the Introduction or Methods and revisited in the Discussion to bolster confidence in interpretation.

In the Introduction, we referenced previous work by Beyene et al. (2019) that validated that nIRCat signal changes in the striatum are specific to dopamine. "Because the striatum is densely innervated by nigrostriatal and mesoaccumbal dopaminergic neurons and lacks innervation from neurons that release norepinephrine, it enables study of evoked dopamine release in this region (Beyene et al., 2019)." (Lines 73 - 76) We can cite more information if needed.

4. Potential DA release and Tau_off Correlations In Figure 5, there appears to be a relationship between reduced release site density, peak dF/F, and increased tau_off. If individual-level correlations exist across slices or mice, these should be quantified and interpreted.

Based on these comments, we have added a new extended data figure where we report correlations between release site density and ∆F/F0 per active release site in dorsal striatum but not NAc. We also mention these findings in the Results, and the full statistics are also reported in the Extended Data Statistical Table. "In a final exploration of our data, we tested for correlations between release site density, ∆F/F0 per active release site, and toff obtained from each slice in each region and treatment group. We found positive correlations between release site density and ∆F/F0 per active release site in dorsal striatum (DMS and DLS) for both sham and GDX groups..." (Lines 360 - 377) 5. Developmental Timing Clarity The manuscript should clearly define the timing of puberty and adolescence in M. spicilegus. While P25-28 are treated as prepubertal, and P60-70 are described as either 'late adolescence' (Line 68) or 'adult' (Line 139), these classifications should be supported with developmental data or appropriate citations.

As pubertal timing is not yet clearly defined in Mus spicilegus and our T data are variable we state: we "estimate P60-70 is a late adolescent or cusp of early adulthood period in this species when reared on 12h:12h photoperiod in the lab environment" (Lines 259 - 261) and we now also use "late adolescence/early adulthood period" to indicate stage at this cusp in other sections.

See also above to Rev 1. We are happy to discuss this decision further. We hope to shed more light on the maturation of these mice in future research using photoperiod manipulations and longitudinal hormone sampling.

Minor Points: Consider reorganizing the Discussion so that references to photoperiod (Line 279 - 280) and ecological traits (Lines 344-352) of M. spicilegus are foregrounded and more clearly linked to the experimental findings.

We reorganized our Discussion to follow this suggestion. "Viewing these physiological and behavioral studies of prepubertal GDX from a neuroethological perspective may help us understand how pubertal processes could drive changes in behavior like dispersal, territory identification, and/or mate seeking (Wright et al., 2020; Lin and Wilbrecht, 2022). Our data are taken from Mus spicilegus, a wild-derived mouse known to disperse from their natal sites..." (Lines 439 - 453) Statistics:

Effect Sizes and P-values: The manuscript should report effect sizes and exact p-values (not just thresholds), particularly for comparisons in Figures 3d, 4c, and 4d. Given similar trends of GDX effects across striatal regions, reliance on a p < 0.05 threshold may not robustly support the binary conclusion that mesoaccumbal circuits are unaffected. Particularly, the n for NAc is smaller than for DLS and DMS, potentially limiting power to detect effects. A discussion of this limitation should be included.

Thanks for prompting us to revisit our statistical analyses. In revision, we moved to use linear mixed effects models to account for non-independence across slices from same animals. We also added exact p-values and effect sizes in the Results text as well as the Extended Data Statistical Table. We also added the limitation that the sample size for NAc is smaller than for DLS and DMS in the Discussion.

We think these changes make this manuscript much improved.

Figure 1a Variability: The SEM for P60 is particularly large. Plotting individual cage means (4 cages in total) would help readers assess biological variability. The source of this heterogeneity should be discussed.

As the fecal testosterone data are preliminary, we briefly addressed the variability of fecal testosterone levels in the Results section. We updated the graph in Figure 1 to show the individual cage means. "Age at gonadectomy was chosen based on data from Mus musculus (Piekarski et al., 2017) and a preliminary study of fecal testosterone (T) of M. spicilegus in which we found a ~ 50% rise in T between P30-90 with high variability between cages (Figure 1a)." (Lines 254 - 257) Image Analysis Software:

Based on the method description, the choice of a 7 mm × 7 mm grid to measure release sites needs justification. Was this resolution optimized empirically or chosen based on prior studies? Could alternative scales change the interpretation of release density? In Results, we provide a justification for the choice of a 7 mm × 7 mm grid. "A previous study observed that electrically evoked dopamine diffuses into the surrounding extracellular space, with over 95% of release sites exhibiting diffusion restricted within 7 mm (Beyene et al., 2019). A grid size of 7 mm was therefore chosen to capture the relevant spatial extent of dopamine diffusion." (Lines 270 - 274) We also added new analyses showing that smaller grid sizes produced comparable results, while results on some metrics washed out with larger grids (10 mm). These results can be found in Extended Data Figure 1-1 and Figure 1-2, with full statistical reporting in the Extended Data Statistical Table. We think this adds valuable information to the new field of imaging 'hotspots.' Thanks for these ideas and your time. We think this improves the manuscript immensely.
