# Supplementary material for: Absence of Testes at Puberty Impacts Functional Development of Nigrostriatal But Not Mesoaccumbal Dopamine Terminals in a Wild-Derived Mouse
Source: eNeuro. 2026 Jan 7;13(1):ENEURO.0212-25.2025. doi: 10.1523/ENEURO.0212-25.2025 (PMC12807560; doi:10.1523/ENEURO.0212-25.2025)
Supplement: Figure 1-1 — Group differences between treatment groups were replicated using a smaller 4 × 4 μm ROI grid size than 7 × 7 μm, while some group differences were no longer significant using a larger 10 × 10 μm ROI grid size. When the ROI grid size was set at 4 × 4 μm resolution comparisons of evoked dopamine release, release site density, and τoff replicated the results obtained applying a 7 × 7 μm resolution grid (see results in the main manuscript). However, when ROI sizes were set at 10 × 10 μm resolution some group differences were no longer significant (DMS peak ΔF/F0 and DLS τoff). Changes with larger grid size may be due to loss of detection of some signals when averaged with non-responsive pixels within the ROI. Linear mixed effects model and effect size calculated using Cohen’s f2. *p < 0.05. Download Figure 1-1, DOCX file. [file eneuro-13-ENEURO.0212-25.2025-s003.docx]

|  | **Parameter** | **Region** | **Effect size** | **Test value** | ***p*-value** | **Power (95% C.I. of diff)** |
| --- | --- | --- | --- | --- | --- | --- |
| ROI grid size 4 μm x 4 μm | Peak ∆F/F_0_ per release site | DLS | 𝑓^2^ = 0.205 | t_10_ = 2.310 | 0.044* | [0.001, 0.031] |
|  |  | DMS | 𝑓^2^ = 0.231 | t_10_ = 2.450 | 0.034* | [0.001, 0.025] |
|  |  | NAc | 𝑓^2^ = 0.060 | t_9_ = 1.144 | 0.282 | [-0.005, 0.014] |
|  | Release site density (%) | DLS | 𝑓^2^ = 0.322 | t_10_ = 2.896 | 0.016* | [5.668, 43.512] |
|  |  | DMS | 𝑓^2^ = 0.074 | t_10_ = 1.383 | 0.197 | [-7.215, 30.825] |
|  |  | NAc | 𝑓^2^ = 0.071 | t_9_ = 1.252 | 0.242 | [-4.236, 14.736] |
|  | τ_off_ | DLS | 𝑓^2^ = 0.368 | t_10_ = -3.093 | 0.011* | [-1.436, -0.233] |
|  |  | DMS | 𝑓^2^ = 0.152 | t_10_ = -1.989 | 0.075 | [-1.210, 0.069] |
|  |  | NAc | 𝑓^2^ = 0.052 | t_9_ = -1.074 | 0.311 | [-1.234, 0.439] |
| ROI grid size 10 μm x 10 μm | Peak ∆F/F_0_ per release site | DLS | 𝑓^2^ = 0.227 | t_10_ = 2.430 | 0.035* | [0.002, 0.035] |
|  |  | DMS | 𝑓^2^ = 0.151 | t_10_ = 1.981 | 0.076 | [-0.001, 0.025] |
|  |  | NAc | 𝑓^2^ = 0.127 | t_9_ = 1.674 | 0.128 | [-0.002, 0.014] |
|  | Release site density (%) | DLS | 𝑓^2^ = 0.215 | t_10_ = 2.364 | 0.040* | [1.384, 46.903] |
|  |  | DMS | 𝑓^2^ = 0.066 | t_10_ = 1.312 | 0.219 | [-9.446, 36.515] |
|  |  | NAc | 𝑓^2^ = 0.075 | t_9_ = 1.280 | 0.232 | [-5.514, 19.897] |
|  | τ_off_ | DLS | 𝑓^2^ = 0.023 | t_10_ = -0.781 | 0.453 | [-1.478, 0.710] |
|  |  | DMS | 𝑓^2^ = 0.291 | t_10_ = -2.753 | 0.020* | [-1.697, -0.179] |
|  |  | NAc | 𝑓^2^ = 0.042 | t_9_ = 0.966 | 0.360 | [-1.637, 4.074] |
