# Supplementary figures and images for: Absence of Testes at Puberty Impacts Functional Development of Nigrostriatal But Not Mesoaccumbal Dopamine Terminals in a Wild-Derived Mouse
Source: eNeuro. 2026 Jan 7;13(1):ENEURO.0212-25.2025. doi: 10.1523/ENEURO.0212-25.2025 (PMC12807560; doi:10.1523/ENEURO.0212-25.2025)

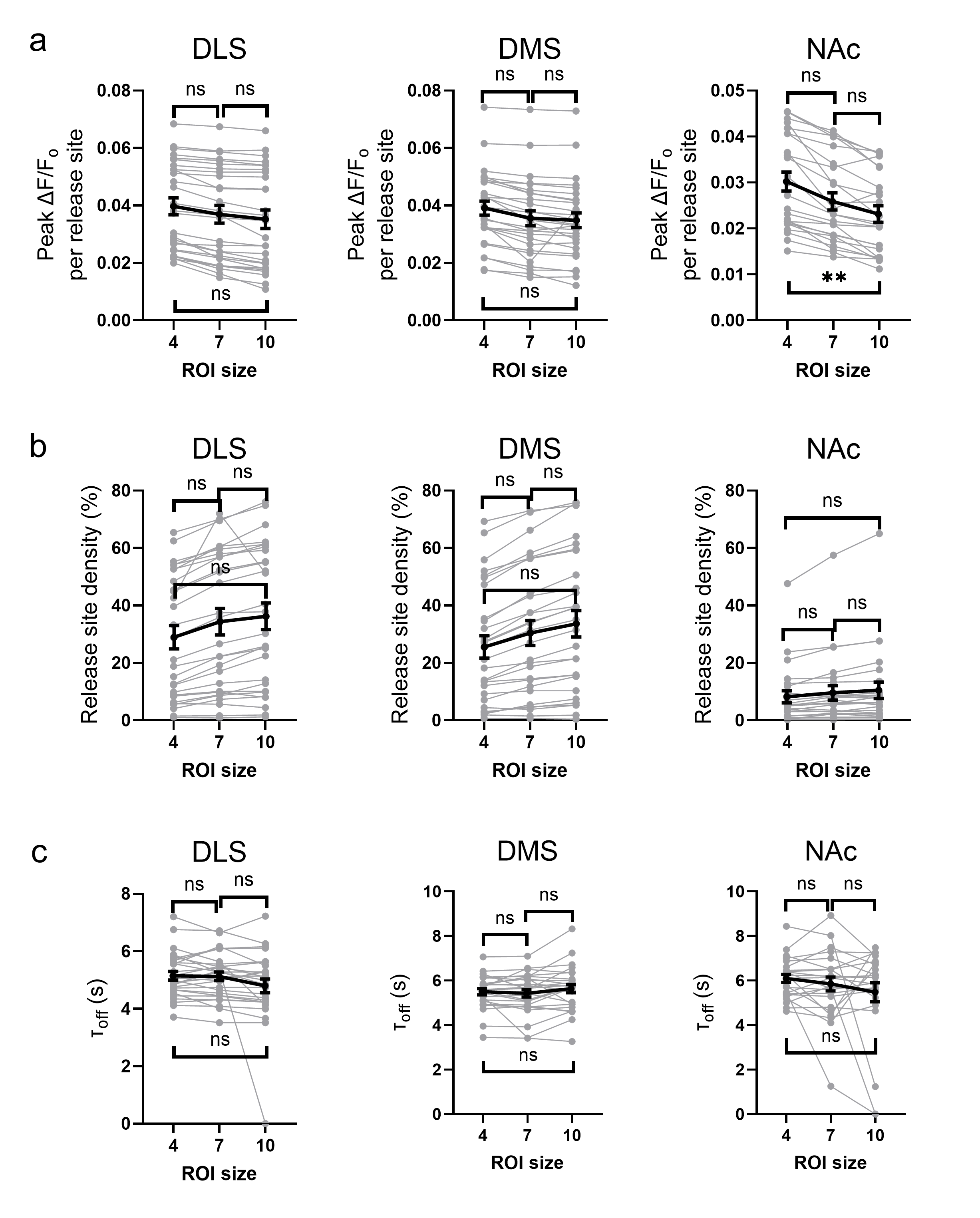

Supplement: Figure 1-2 — Evoked dopamine release, release site density, and reuptake related metric were comparable across regions and treatment groups when ROI sizes were set at smaller resolution. Comparison of (a) peak ΔF/F0 per release site, (b) release site density, and (c) τoff between ROI sizes of 7 × 7 μm, 4 × 4 μm, and 10 × 10 μm for each region. Plots of data from all mice (a-c) showed few significant changes in mean data values when analyzed using three different grid sizes (4, 7, 10 μm). Linear mixed effects model followed by post-hoc analysis with Tukey’s test. **p < 0.01. Error bars represent the SEM. Download Figure 1-2, TIF file. [file eneuro-13-ENEURO.0212-25.2025-s001.tif]

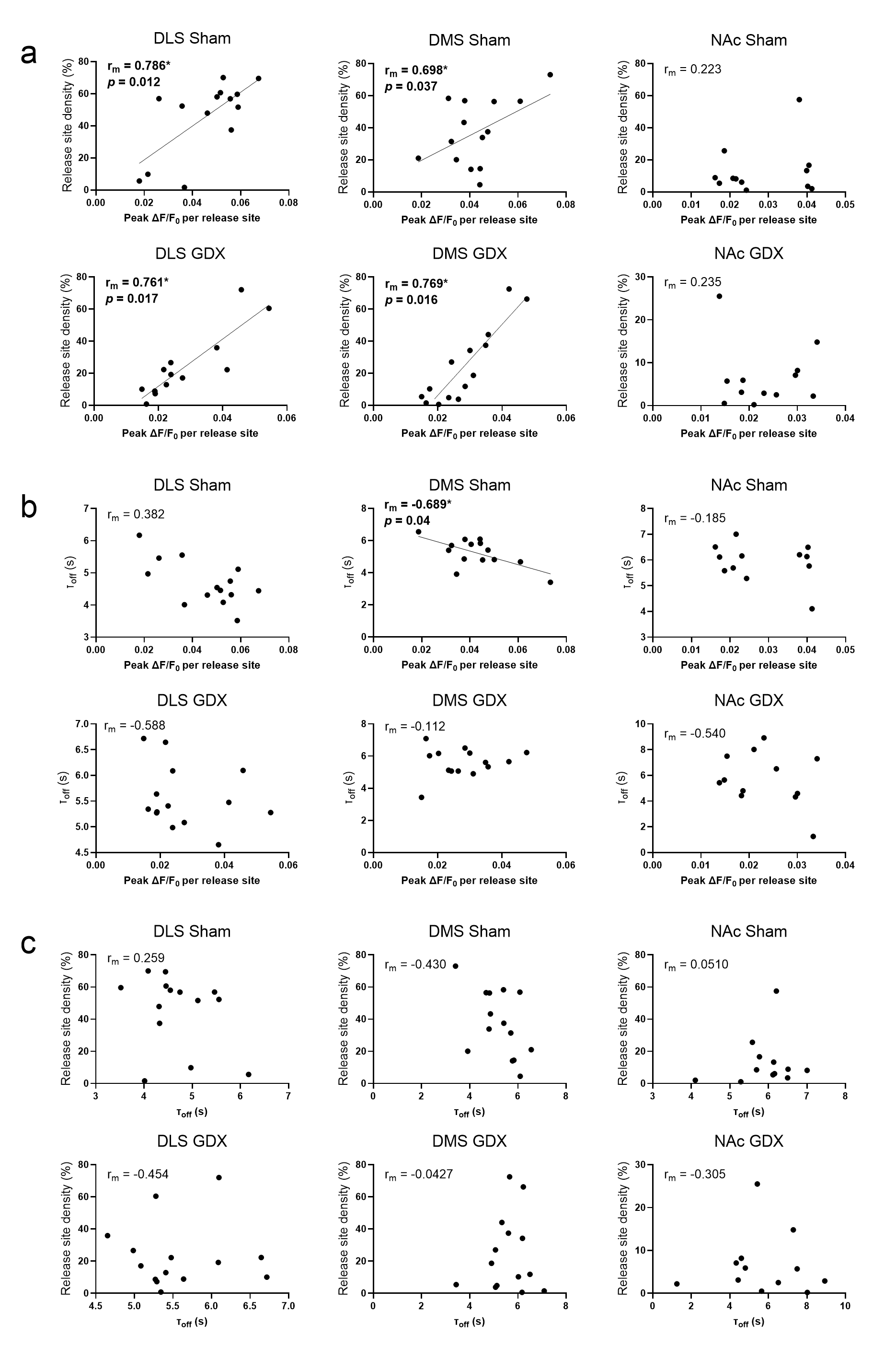

Supplement: Figure 5-1 — Evoked dopamine release and release site density were positively correlated in the dorsal striatum, but not NAc. (a) Comparison between peak ΔF/F0 per release site and release site density, (b) peak ΔF/F0 per release site and τoff, and (c) τoff and release site density for each region and treatment group. Repeated measures correlation. *p < 0.05. These analyses were exploratory and p-values were not corrected for multiple comparisons. Download Figure 5-1, TIF file. [file eneuro-13-ENEURO.0212-25.2025-s002.tif]
