# Supplementary material for: Absence of Testes at Puberty Impacts Functional Development of Nigrostriatal But Not Mesoaccumbal Dopamine Terminals in a Wild-Derived Mouse
Source: eNeuro. 2026 Jan 7;13(1):ENEURO.0212-25.2025. doi: 10.1523/ENEURO.0212-25.2025 (PMC12807560; doi:10.1523/ENEURO.0212-25.2025)
Supplement: Table 1-2 — Statistical Table. Download Figure 1-2, DOCX file. [file eneuro-13-ENEURO.0212-25.2025-s004.docx]

**Extended Data Figure 1-2. Statistical Table**

|  | **Figure** | **Data structure** | **Type of test** | **Effect size** | **Test value** | ***p*-value** | **Power (95% C.I. of diff)** |
| --- | --- | --- | --- | --- | --- | --- | --- |
| *a* | 2c | Hierarchical data | Linear mixed effects model; Cohen’s 𝑓^2^ | 𝑓^2^ = 0.226 | t_10_ = 2.427 | 0.036 | [0.001, 0.034] |
| *b* | 2d | Hierarchical data | Linear mixed effects model; Cohen’s 𝑓^2^ | 𝑓^2^ = 0.206 | t_10_ = 2.315 | 0.043 | [0.878, 46.168] |
| *c* | 2e | Hierarchical data | Linear mixed effects model; Cohen’s 𝑓^2^ | 𝑓^2^ = 0.306 | t_10_ = -2.821 | 0.018 | [-1.517, -0.178] |
| *d* | 3c | Hierarchical data | Linear mixed effects model; Cohen’s 𝑓^2^ | 𝑓^2^ = 0.205 | t_10_ = 2.309 | 0.044 | [0.000481, 0.027] |
| *e* | 3d | Hierarchical data | Linear mixed effects model; Cohen’s 𝑓^2^ | 𝑓^2^ = 0.071 | t_10_ = 1.356 | 0.205 | [-8.366, 34.39] |
| *f* | 3e | Hierarchical data | Linear mixed effects model; Cohen’s 𝑓^2^ | 𝑓^2^ = 0.025 | t_10_ = -0.807 | 0.439 | [-1.156, 0.542] |
| *g* | 4c | Hierarchical data | Linear mixed effects model; Cohen’s 𝑓^2^ | 𝑓^2^ = 0.095 | t_9_ = 1.448 | 0.182 | [-0.003, 0.014] |
| *h* | 4d | Hierarchical data | Linear mixed effects model; Cohen’s 𝑓^2^ | 𝑓^2^ = 0.078 | t_9_ = 1.314 | 0.221 | [-4.698, 17.714] |
| *i* | 4e | Hierarchical data | Linear mixed effects model; Cohen’s 𝑓^2^ | 𝑓^2^ = 0.004 | t_9_ = 0.305 | 0.767 | [-1.246, 1.634] |
| *j* | 5a  (*DMS - DLS*) | Hierarchical data | Linear mixed effects model; Tukey’s *post-hoc* test; η^2^_p_ and Hedges’ *g* for pairwise comparisons | η^2^_p_ = 0.40  95% CI [0.16, 1.00]  *g* = -0.179 | z = -0.635 | 0.801 | [-0.012, 0.007] |
| *k* | 5a  (*NAc - DLS*) |  |  | η^2^_p_ = 0.40  95% CI [0.16, 1.00]  *g* = -1.23 | z = -4.297 | 0.0000489 | [-0.029, -0.009] |
| *l* | 5a  (NAc - DMS) |  |  | η^2^_p_ = 0.40  95% CI [0.16, 1.00]  *g* = -1.15 | z = -3.695 | 0.000663 | [-0.027, -0.006] |
| *m* | 5b  (*DMS - DLS*) | Hierarchical data | Linear mixed effects model; Tukey’s *post-hoc* test; η^2^_p_ and Hedges’ *g* for pairwise comparisons | η^2^_p_ = 0.36  95% CI [0.13, 1.00]  *g* = -0.367 | z = -1.116 | 0.504 | [-25.767, 9.138] |
| *n* | 5b  (*NAc - DLS*) |  |  | η^2^_p_ = 0.36  95% CI [0.13, 1.00]  *g* = -1.57 | z = -4.199 | 0.0000675 | [-50.924, -14.445] |
| *o* | 5b  (*NAc - DMS*) |  |  | η^2^_p_ = 0.36  95% CI [0.13, 1.00]  *g* = -1.26 | z = -3.131 | 0.005 | [-42.610, -6.131] |
| *p* | 5c  (*DMS - DLS*) | Hierarchical data | Linear mixed effects model; Tukey’s *post-hoc* test; η^2^_p_ and Hedges’ *g* for pairwise comparisons | η^2^_p_ = 0.34  95% CI [0.12, 1.00]  *g* = 0.667 | z = 1.912 | 0.135 | [-0.123, 1.213] |
| *q* | 5c  (*NAc - DLS*) |  |  | η^2^_p_ = 0.34  95% CI [0.12, 1.00]  *g* = 1.66 | z = 4.159 | 0.000102 | [0.541, 1.938] |
| *r* | 5c  (*NAc - DMS*) |  |  | η^2^_p_ = 0.34  95% CI [0.12, 1.00]  *g* = 0.810 | z = 2.329 | 0.052 | [-0.004, 1.393] |
| *s* | 5d  (*DMS - DLS*) | Hierarchical data | Linear mixed effects model; Tukey’s *post-hoc* test; η^2^_p_ and Hedges’ *g* for pairwise comparisons | η^2^_p_ = 0.08  95% CI [0.00, 1.00]  *g* = 0.040 | z = 0.141 | 0.989 | [-0.007, 0.008] |
| *t* | 5d  (*NAc - DLS*) |  |  | η^2^_p_ = 0.08  95% CI [0.00, 1.00]  *g* = -0.423 | z = -1.383 | 0.350 | [-0.012, 0.003] |
| *u* | 5d  (*NAc - DMS*) |  |  | η^2^_p_ = 0.08  95% CI [0.00, 1.00]  *g* = -0.552 | z = -1.518 | 0.282 | [-0.013, 0.003] |
| *v* | 5e  (*DMS - DLS*) | Hierarchical data | Linear mixed effects model; Tukey’s *post-hoc* test; η^2^_p_ and Hedges’ *g* for pairwise comparisons | η^2^_p_ = 0.21  95% CI [0.02, 1.00]  *g* = 0.043 | z = 0.152 | 0.987 | [-14.354, 16.339] |
| *w* | 5e  (*NAc - DLS*) |  |  | η^2^_p_ = 0.21  95% CI [0.02, 1.00]  *g* = -1.02 | z = -2.488 | 0.034 | [-33.042, -0.989] |
| *x* | 5e  (*NAc - DMS*) |  |  | η^2^_p_ = 0.21  95% CI [0.02, 1.00]  *g* = -0.938 | z = -2.633 | 0.023 | [-34.035, -1.982] |
| *y* | 5f  (*DMS - DLS*) | Hierarchical data | Linear mixed effects model; Tukey’s *post-hoc* test; η^2^_p_ and Hedges’ *g* for pairwise comparisons | η^2^_p_ = 0.003  95% CI [0.00, 1.00]  *g* = 0.040 | z = 0.064 | 0.998 | [-1.109, 1.172] |
| *z* | 5f  (*NAc - DLS*) |  |  | η^2^_p_ = 0.003  95% CI [0.00, 1.00]  *g* = 0.104 | z = 0.300 | 0.952 | [-1.036, 1.340] |
| *ab* | 5f  (*NAc - DMS*) |  |  | η^2^_p_ = 0.003  95% CI [0.00, 1.00]  *g* = 0.079 | z = 0.238 | 0.969 | [-1.067, 1.309] |
|  | 1-2a  (*DLS*) | Hierarchical data | Linear mixed effects model; Tukey’s *post-hoc* test; η^2^_p_ and Hedges’ *g* for pairwise comparisons | η^2^_p_ = 0.06  95% CI [0.00, 1.00]  (*7 - 4*)  *g* = -0.173  (*10 - 4*)  *g* = -0.272  (*10 - 7*)  *g* = -0.100 | (*7 - 4*)  z = -1.259  (*10 - 4*)  z = -2.029  (*10 - 7*)  z = -0.770 | (*7 - 4*)  0.418  (*10 - 4*)  0.105  (*10 - 7*)  0.721 | (*7 - 4*)  [-0.008, 0.002]  (*10 - 4*)  [-0.010, 0.001]  (*10 - 7*)  [-0.007, 0.003] |
|  | 1-2a  (*DMS*) |  |  | η^2^_p_ = 0.07  95% CI [0.00, 1.00]  (*7 - 4*)  *g* = -0.260  (*10 - 4*)  *g* = -0.314  (*10 - 7*)  *g* = -0.052 | (*7 - 4*)  z = -1.803  (*10 - 4*)  z = -2.174  (*10 - 7*)  z = -0.371 | (*7 - 4*)  0.169  (*10 - 4*)  0.076  (*10 - 7*)  0.927 | (*7 - 4*)  [-0.008, 0.001]  (*10 - 4*)  [-0.009, 0.000332]  (*10 - 7*)  [-0.005, 0.004] |
|  | 1-2a  (*NAc*) |  |  | η^2^_p_ = 0.13  95% CI [0.02, 1.00]  (*7 - 4*)  *g* = -0.437  (*10 - 4*)  *g* = -0.730  (*10 - 7*)  *g* = -0.300 | (*7 - 4*)  z = -1.815  (*10 - 4*)  z = -2.972  (*10 - 7*)  z = -1.157 | (*7 - 4*)  0.165  (*10 - 4*)  0.008  (*10 - 7*)  0.479 | (*7 - 4*)  [-0.010, 0.001]  (*10 - 4*)  [-0.013, -0.001]  (*10 - 7*)  [-0.008, 0.003] |
|  | 1-2b  (*DLS)* | Hierarchical data | Linear mixed effects model; Tukey’s *post-hoc* test; η^2^_p_ and Hedges’ *g* for pairwise comparisons | η^2^_p_ = 0.05  95% CI [0.00, 1.00]  (*7 - 4*)  *g* = 0.232  (*10 - 4*)  *g* = 0.311  (*10 - 7*)  *g* = 0.077 | (*7 - 4*)  z = 1.405  (*10 - 4*)  z = 1.898  (*10 - 7*)  z = 0.493 | (*7 - 4*)  0.338  (*10 - 4*)  0.139  (*10 - 7*)  0.875 | (*7 - 4*)  [-3.609, 14.416]  (*10 - 4*)  [-1.712, 16.312]  (*10 - 7*)  [-7.116, 10.909] |
|  | 1-2b  (*DMS)* |  |  | η^2^_p_ = 0.04  95% CI [0.00, 1.00]  (*7 - 4*)  *g* = 0.219  (*10 - 4*)  *g* = 0.351  (*10 - 7*)  *g* = 0.133 | (*7 - 4*)  z = 1.083  (*10 - 4*)  z = 1.796  (*10 - 7*)  z = 0.713 | (*7 - 4*)  0.525  (*10 - 4*)  0.171  (*10 - 7*)  0.756 | (*7 - 4*)  [-5.641, 15.334]  (*10 - 4*)  [-2.452, 18.523]  (*10 - 7*)  [-7.298, 13.677] |
|  | 1-2b  (*NAc)* |  |  | η^2^_p_ = 0.010  95% CI [0.00, 1.00]  (*7 - 4*)  *g* = 0.118  (*10 - 4*)  *g* = 0.180  (*10 - 7*)  *g* = 0.067 | (*7 - 4*)  z = 0.458  (*10 - 4*)  z = 0.758  (*10 - 7*)  z = 0.300 | (*7 - 4*)  0.891  (*10 - 4*)  0.729  (*10 - 7*)  0.952 | (*7 - 4*)  [-5.607, 8.332]  (*10 - 4*)  [-4.716, 9.224]  (*10 - 7*)  [-6.078, 7.861] |
|  | 1-2c  (*DLS)* | Hierarchical data | Linear mixed effects model; Tukey’s *post-hoc* test; η^2^_p_ and Hedges’ *g* for pairwise comparisons | η^2^_p_ = 0.02  95% CI [0.00, 1.00]  (*7 - 4*)  *g* = -0.027  (*10 - 4*)  *g* = -0.207  (*10 - 7*)  *g* = -0.181 | (*7 - 4*)  z = -0.153  (*10 - 4*)  z = -1.037  (*10 - 7*)  z = -0.885 | (*7 - 4*)  0.987  (*10 - 4*)  0.554  (*10 - 7*)  0.650 | (*7 - 4*)  [-0.343, 0.301]  (*10 - 4*)  [-0.469, 0.181]  (*10 - 7*)  [-0.448, 0.202] |
|  | 1-2c  (*DMS)* |  |  | η^2^_p_ = 0.02  95% CI [0.00, 1.00]  (*7 - 4*)  *g* = -0.088  (*10 - 4*)  *g* = 0.154  (*10 - 7*)  *g* = 0.220 | (*7 - 4*)  z = -0.405  (*10 - 4*)  z = 0.755  (*10 - 7*)  z = 1.160 | (*7 - 4*)  0.914  (*10 - 4*)  0.731  (*10 - 7*)  0.477 | (*7 - 4*)  [-0.494, 0.349]  (*10 - 4*)  [-0.286, 0.557]  (*10 - 7*)  [-0.213, 0.630] |
|  | 1-2c  (*NAc)* |  |  | η^2^_p_ = 0.008  95% CI [0.00, 1.00]  (*7 - 4*)  *g* = -0.191  (*10 - 4*)  *g* = -0.109  (*10 - 7*)  *g* = 0.083 | (*7 - 4*)  z = -0.676  (*10 - 4*)  z = -0.360  (*10 - 7*)  z = 0.301 | (*7 - 4*)  0.778  (*10 - 4*)  0.931  (*10 - 7*)  0.951 | (*7 - 4*)  [-1.094, 0.604]  (*10 - 4*)  [-1.002, 0.735]  (*10 - 7*)  [-0.757, 0.980] |
| *ac* | 5-1a  (*DLS sham*) | Hierarchical data | Repeated measures correlation | r_m_ = 0.786 | – | 0.012 | [0.254, 0.953] |
| *ad* | 5-1a  (*DMS sham*) |  |  | r_m_ = 0.698 | – | 0.037 | [0.063, 0.931] |
| *ae* | 5-1a  (*NAc sham*) |  |  | r_m_ = 0.223 | – | 0.596 | [-0.572, 0.802] |
| *af* | 5-1a  (*DLS GDX*) |  |  | r_m_ = 0.761 | – | 0.017 | [0.196, 0.947] |
| *ag* | 5-1a  (*DMS GDX*) |  |  | r_m_ = 0.769 | – | 0.016 | [0.213, 0.949] |
| *ah* | 5-1a  (*NAc GDX*) |  |  | r_m_ = 0.235 | – | 0.613 | [-0.630, 0.839] |
| *ai* | 5-1b  (*DLS sham*) | Hierarchical data | Repeated measures correlation | r_m_ = 0.382 | – | 0.310 | [-0.378, 0.834] |
| *aj* | 5-1b  (*DMS sham*) |  |  | r_m_ = -0.689 | – | 0.040 | [-0.928, -0.045] |
| *ak* | 5-1b  (*NAc sham*) |  |  | r_m_ = -0.185 | – | 0.661 | [-0.787, 0.597] |
| *al* | 5-1b  (*DLS GDX*) |  |  | r_m_ = -0.588 | – | 0.096 | [-0.900, 0.125] |
| *am* | 5-1b  (*DMS GDX*) |  |  | r_m_ = -0.112 | – | 0.774 | [-0.722, 0.597] |
| *an* | 5-1b  (*NAc GDX*) |  |  | r_m_ = -0.540 | – | 0.211 | [-0.919, 0.359] |
| *ao* | 5-1c  (*DLS sham*) | Hierarchical data | Repeated measures correlation | r_m_ = 0.259 | – | 0.501 | [-0.489, 0.788] |
| *ap* | 5-1c  (*DMS sham*) |  |  | r_m_ = -0.430 | – | 0.248 | [-0.851, 0.327] |
| *aq* | 5-1c  (*NAc sham*) |  |  | r_m_ = 0.051 | – | 0.905 | [-0.678, 0.729] |
| *ar* | 5-1c  (*DLS GDX*) |  |  | r_m_ = -0.454 | – | 0.220 | [-0.859, 0.301] |
| *as* | 5-1c  (*DMS GDX*) |  |  | r_m_ = -0.043 | – | 0.913 | [-0.687, 0.640] |
| *at* | 5-1c  (*NAc GDX*) |  |  | r_m_ = -0.305 | – | 0.506 | [-0.860, 0.582] |
